# Beyond d‐Band Catalysis: A Critical Review and Descriptor Framework for Rare‐Earth Engineering in Lithium–Sulfur Batteries

**DOI:** 10.1002/advs.75890

**Published:** 2026-06-01

**Authors:** Fan Wang, Shihzad Shakil, Guozhi Wu, Jiarui Huang, Hanhui Lei, Terence Xiaoteng Liu

**Affiliations:** ^1^ School of Materials Science and Engineering Tongling University Tongling Anhui P. R. China; ^2^ Anhui Engineering Research Center of Highly Reactive Micro‐Nano Powders School of Materials and Environmental Engineering Chizhou University Chizhou P. R. China; ^3^ Key Laboratory of Functional Molecular Solids College of Chemistry and Materials Science Ministry of Education Anhui Normal University Wuhu P. R. China; ^4^ School of Engineering Physics and Mathematics Faculty of Science and Environment Northumbria University Newcastle upon Tyne UK

**Keywords:** descriptor‐guided design, f‐orbital catalysis, lithium–sulfur batteries, oxygen vacancy engineering, polysulfide regulation, RARE‐earth elements (REEs), single‐atom catalysts

## Abstract

Rare earth elements (REEs) have emerged as a distinctive class of functional materials for lithium–sulfur (Li–S) batteries, offering catalytic behavior that extends beyond the conventional d‐band paradigm of transition‐metal systems. Their localized 4f orbitals, variable oxidation states, strong Lewis acidity, and defect chemistry enable multifunctional regulation of polysulfide adsorption and redox conversion. They also enhance interfacial stability. This review evaluates REE‐based materials across cathodes, separators, electrolyte/additive systems, and integrated cell architectures, with emphasis on how REEs function as redox mediators, polar anchors, and electronic/ionic interface modulators. Some credible advances arise not from isolated material effects, but from conductive integration, balanced adsorption–conversion, and coordinated deployment across multiple cell components. By benchmarking reported systems against practical metrics, including high sulfur loading, areal capacity, rate capability, and pouch‐cell relevance, we identify promising REE‐enabled architectures while also highlighting persistent limitations in mechanistic validation and reporting consistency. We further argue that rational progress in this field requires a descriptor‐guided framework tailored to 4f chemistry, in which crystal‐field splitting, electronegativity, f–d hybridization, oxygen vacancy concentration, and Lewis acidity collectively govern catalytic behavior. Finally, we outline future directions centered on underexplored lanthanides, operando and multiscale characterization, hybrid REE/transition‐metal designs, and circular‐economy strategies for sustainable deployment.

## Graphical abstract

Rare‐earth catalysts regulate lithium–sulfur battery chemistry through f‐orbital–mediated interactions, enabling simultaneous polysulfide adsorption and catalytic conversion on conductive carbon hosts. This synergistic control suppresses the shuttle effect, accelerates redox kinetics, and guides stable Li_2_S nucleation, providing a mechanistic framework for improved Li–S battery performance.

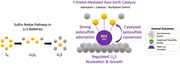

## 1 Introduction

### 1.1 Lithium‐Sulfur (Li–S) Batteries: Promise and Persistent Challenges

The accelerating global transition toward renewable energy systems and electric mobility has intensified the search for energy storage technologies that surpass the limitations of conventional lithium‐ion batteries (LIBs). Among emerging candidates, Li–S batteries are widely regarded as one of the most promising next‐generation systems because of their exceptionally high theoretical capacity, material abundance, and potential cost advantages [1]. Li–S batteries offer a theoretical specific capacity of 1675 mAh g^−1^ and an energy density approaching 2600 Wh kg^−1^. This is approximately five times greater than that of conventional intercalation‐based LIBs, making them attractive for long‐range electric vehicles and grid‐scale storage applications [2]. Moreover, sulfur is earth‐abundant, environmentally benign, and inexpensive, providing a sustainable alternative to cobalt‐ and nickel‐dependent cathode chemistries used in LIBs [3]. Despite these advantages, the practical commercialization of Li–S batteries remain constrained by several intrinsic challenges rooted in sulfur electrochemistry. However, these challenges are not independent; rather, they are strongly coupled through the complex interplay between polysulfide transport, reaction kinetics, and electrode structure. This coupling implies that isolated material‐level solutions are often insufficient, necessitating integrated strategies that simultaneously address transport, catalysis, and structural stability.

A primary limitation is the extremely low electrical conductivity of both elemental sulfur (S_8_) and the final discharge product lithium sulfide (Li_2_S). This insulating nature necessitates the addition of large quantities of conductive additives, reducing overall energy density and complicating electrode fabrication. Furthermore, sluggish ionic transport within Li_2_S impedes complete sulfur utilization at high rates, leading to capacity loss and reduced power performance [4]. A second and more complex challenge arises from the multistep redox chemistry of sulfur. During discharge, sulfur undergoes sequential reduction reactions. These reactions generate soluble lithium polysulfide intermediates (Li_2_S_x_, 4 ≤ x ≤ 8) [5]. These species readily dissolve into the electrolyte and migrate toward the lithium metal anode, a phenomenon known as the polysulfide shuttle effect [6]. Although numerous materials have been developed to suppress this shuttle behavior, many rely primarily on strong adsorption, which can inadvertently impede the reversibility of sulfur redox reactions by immobilizing intermediate species. At the anode, polysulfides are reduced to insoluble Li_2_S/Li_2_S_2_ and can diffuse back to the cathode. This establishes parasitic redox cycles that cause active material loss, low Coulombic efficiency, severe self‐discharge, and lithium anode corrosion [7].

**FIGURE 1:**
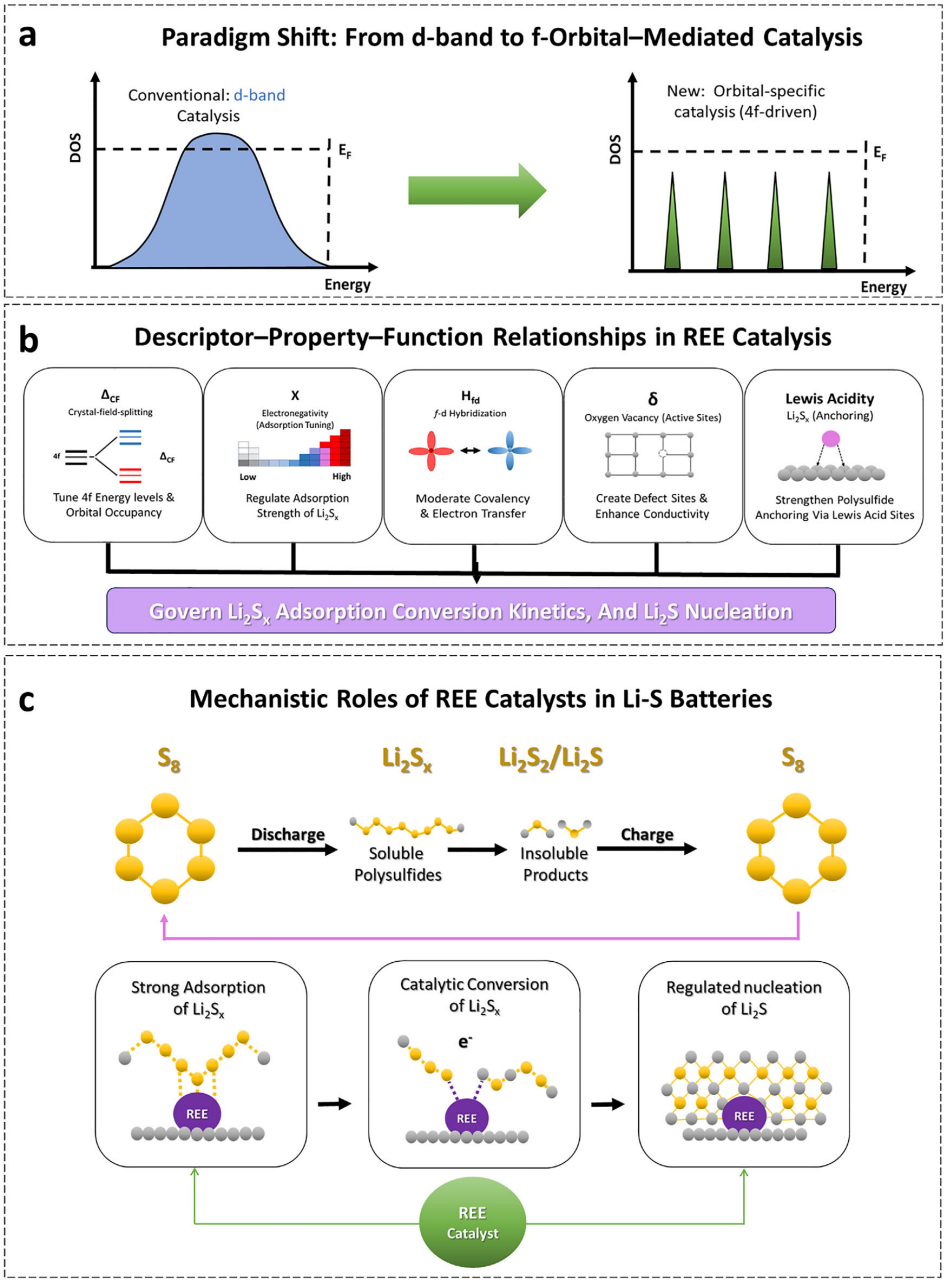
Conceptual framework of descriptor‐guided rare‐earth catalysis in Li–S batteries. (a) Paradigm shift from conventional d‐band catalysis to f‐orbital–mediated catalysis, highlighting the transition from delocalized d‐states to localized 4f electronic states near the Fermi level. (b) Descriptor–property–function relationships in rare‐earth systems, where key descriptors (crystal‐field splitting, electronegativity, f–d hybridization, oxygen vacancies, and Lewis acidity) collectively control polysulfide adsorption, redox kinetics, and Li_2_S nucleation. (c) Mechanistic roles of rare‐earth catalysts in Li–S batteries, including strong adsorption of Li_2_S_x_ intermediates, catalytic acceleration of sulfur redox conversion, and regulated Li_2_S nucleation, leading to suppressed shuttle effect and improved electrochemical performance.

In addition to electrochemical challenges, Li–S batteries suffer from pronounced mechanical instability. The conversion of sulfur to Li_2_S involves ∼80% volumetric expansion, generating substantial stress within the cathode structure [8]. Repeated expansion and contraction during cycling lead to structural degradation, loss of electrical connectivity, and accelerated capacity fading. These coupled electrochemical and structural processes are illustrated in Figure 1c, which depicts the sulfur redox pathway (S_8_ → Li_2_S_x_ → Li_2_S) together with the key catalytic requirements. Effective catalysts must simultaneously regulate polysulfide adsorption, accelerate redox conversion, and control Li_2_S nucleation. The inability to coordinate these processes under practical conditions is a central limitation of conventional material strategies. These interconnected challenges include electrical insulation, polysulfide shuttle effect, sluggish reaction kinetics, and structural instability. They have driven extensive research into advanced cathode hosts and electrocatalysts designed to stabilize sulfur chemistry and accelerate redox kinetics [9, 10]. Taken together, these challenges reveal a critical limitation of conventional material design strategies: most approaches address individual failure modes but fail to simultaneously regulate polysulfide transport, reaction kinetics, and mechanical stability under practical conditions. Within this context, electrocatalysis has emerged as a promising strategy to regulate sulfur redox chemistry. Among various catalytic materials, REEs have recently attracted growing attention due to their distinctive electronic structures and surface chemistries.

### 1.2 Why Rare Earth Elements? A Unique Functional Perspective

REEs, comprising the lanthanide series along with scandium and yttrium, possess unique physicochemical properties arising from partially filled 4f orbitals and high coordination flexibility [11, 12]. These features have enabled widespread applications in catalysis and energy technologies. Certain REEs exhibit accessible multiple oxidation states, such as Ce^3+^/Ce^4+^. This enables their participation in electron‐transfer processes during sulfur conversion. Cerium dioxide (CeO_2_) is especially notable for its reversible Ce^3+^ ⇌ Ce^4+^ transition and oxygen storage capability, which can promote catalytic polysulfide conversion and improve reaction kinetics [13, 14]. REE oxides possess highly polar surfaces and strong Lewis acidity, enabling strong chemical interactions with negatively charged polysulfide species and suppressing shuttle behavior [15]. REE incorporation can alter adsorption energetics and reduce activation barriers for sulfur redox reactions. Theoretical studies indicate that electronic modulation and orbital interactions can enhance catalytic activity in hybrid systems [16, 17]. However, it remains unclear whether these enhancements arise from intrinsic 4f electronic effects or from more general features such as surface polarity and defect chemistry, which are also present in non‐REE materials.

Transition‐metal catalysts derive activity primarily from d‐orbital electronic structures described by d‐band theory [18]. This distinction is illustrated in Figure 1a, which contrasts conventional d‐band catalysis with f‐orbital–mediated catalysis in rare‐earth systems. While d‐band theory describes adsorption behavior based on the position of delocalized d‐states relative to the Fermi level, the localized 4f orbitals in rare‐earth elements enable electronic modulation through crystal‐field effects and defect‐mediated interactions. This fundamental difference provides an alternative framework for tuning polysulfide conversion pathways.

In contrast, REE chemistry is governed by localized 4f orbitals, which influence bonding through crystal‐field effects and electronic modulation, enabling alternative reaction pathways and stabilization mechanisms [19, 20, 21]. In our view, the key advantage of REEs lies not merely in stronger polysulfide adsorption, but in their ability to modulate local electronic environments through crystal‐field effects and defect‐mediated interactions. This potentially enables reaction pathways that differ fundamentally from conventional d‐band catalysts.

### 1.3 Knowledge Gaps and This Review's Contributions

Critical evaluation of the literature reveals several gaps. These gaps hinder the rational development of REE‐based Li–S materials. First, catalytic mechanisms remain poorly defined, with limited understanding of active sites and reaction pathways. This review provides a critical deconstruction of proposed mechanisms, distinguishing established principles from speculative claims. Second, research has focused predominantly on cerium and lanthanum, leaving mid‐ and heavy‐lanthanides underexplored. We systematically analyze periodic trends to identify promising but neglected candidates.

Third, direct comparisons with transition‐metal catalysts remain scarce, and multifunctional deployment of REEs across cell components is seldom investigated. We establish a comparative framework positioning REE chemistry against conventional d‐band catalysis and synthesize strategies for coordinated REE integration across cathodes, separators, and anodes. Fourth, many studies report performance under idealized laboratory conditions rather than practical sulfur loadings and electrolyte limitations; we critically evaluate reported metrics against commercially relevant benchmarks to separate laboratory curiosities from genuinely viable approaches. Finally, predictive descriptors for REE‐based catalyst design remain underdeveloped. We propose and critically assess a multi‐descriptor framework (crystal field splitting, f–d hybridization strength, oxygen vacancy concentration) that captures the unique physics of 4f orbitals and provides a foundation for rational design. As summarized in Figure 1b, key descriptors such as crystal‐field splitting, electronegativity, f–d hybridization, oxygen vacancy concentration, and Lewis acidity collectively govern polysulfide adsorption, redox kinetics, and Li_2_S nucleation. This descriptor–property–function relationship provides a mechanistic basis for rational catalyst design. Collectively, this review aims not only to summarize existing studies but to critically evaluate their underlying assumptions, identify unresolved challenges, and establish a mechanistically grounded framework for the rational design of REE‐based Li–S systems.

### 1.4 Scope and Organization

This review is structured as follows. Section 2 examines the fundamental chemistry of REEs at electrochemical interfaces, focusing on redox mediation, electronic modulation, and polysulfide interactions. Section 3 surveys REE‐based composite materials carbon hybrids, metal–organic frameworks, oxides, and chalcogenides synthesizing structure–performance relationships and identifying design principles. Section 4 analyzes the functional roles of REEs across cathodes, separators, and anodes, while Section 5 explores integrated, multifunctional deployment strategies.

Section 6 critically evaluates reported performance against commercially relevant metrics, including high sulfur loading and lean electrolyte conditions. Finally, Section 7 synthesizes these insights into a descriptor‐guided roadmap for future research, addressing underexplored lanthanides, advanced characterization needs, and circular economy considerations. By integrating mechanistic understanding with materials design and practical performance evaluation, this review seeks to bridge the gap between fundamental REE chemistry and the development of viable Li–S battery technologies.

## 2 Rare‐Earth Chemistry in Electrochemical Interfaces

The distinctive electrochemical behavior of REEs in Li–S batteries originates from their unique electronic configurations and coordination chemistry. Unlike transition‐metal catalysts, whose activity is typically rationalized through d‐band center theory, lanthanides possess partially filled 4f orbitals that are spatially contracted but can influence interfacial chemistry through mixed valence states (e.g., Ce^3+^/Ce^4+^), crystal‐field effects, local electrostatics, and orbital hybridization with sulfur p‐states. These characteristics enable REE‐based materials to interact with polysulfide species in ways that differ fundamentally from conventional catalysts.

Recent studies (2024–2025) consistently suggest that REE compounds can function as (i) redox mediators, (ii) strong polar adsorption centers for lithium polysulfides (LiPSs), and (iii) electronic/ionic interface modulators, thereby enabling simultaneous suppression of the shuttle effect and acceleration of bidirectional sulfur conversion [22]. However, these roles are frequently inferred from electrochemical performance improvements rather than directly validated through operando or mechanistic investigations. Consequently, the relative contributions of redox mediation, adsorption, and interfacial electronic modulation remain difficult to decouple, leading to ambiguity in identifying the true active sites and dominant catalytic pathways.

### 2.1 Redox Mediators and Catalytic Active Sites

The sulfur reduction and evolution reactions in Li–S batteries involve a multistep cascade: S_8_ → soluble Li_2_S_x_ → insoluble Li_2_S_2_/Li_2_S. Sluggish kinetics and soluble intermediates lead to polarization and the polysulfide shuttle effect. REE‐based catalysts have been proposed to address these limitations through three primary functions: (i) polar anchoring of LiPSs, (ii) catalytic promotion of conversion reactions, and (iii) redox‐active mediation. Importantly, these functions are not mutually exclusive and often coexist within a single material, making it challenging to isolate the dominant mechanism responsible for performance enhancement. Operando X‐ray absorption spectroscopy (XAS) studies on Ce^4+^‐based MOFs revealed that one Ce^4+^ site per Ce_6_ cluster is reduced to Ce^3+^ during catalysis, accompanied by an outward radial shift while maintaining structural integrity [23]. This observation highlights the role of the Ce^4+^/Ce^3+^ redox couple as a dynamic mediator during sulfur conversion. However, since this redox transition involves only a single‐electron process, additional mechanisms are required to facilitate the multi‐electron reactions associated with polysulfide conversion.

Although many mechanistic insights into REE redox behavior originate from systems outside Li–S chemistry, they provide valuable conceptual guidance. For instance, studies have shown that inner‐sphere coordination can dominate electron‐transfer kinetics in Ce‐based systems, while defect‐rich CeO_2_ and Eu‐doped oxides enhance catalytic activity through vacancy‐mediated electronic modulation [24]. These findings suggest that the coordination environment and defect chemistry are critical in determining catalytic behavior. Redox potential alone is insufficient. However, their direct applicability to sulfur redox reactions remains to be conclusively established [25, 26].

**FIGURE 2:**
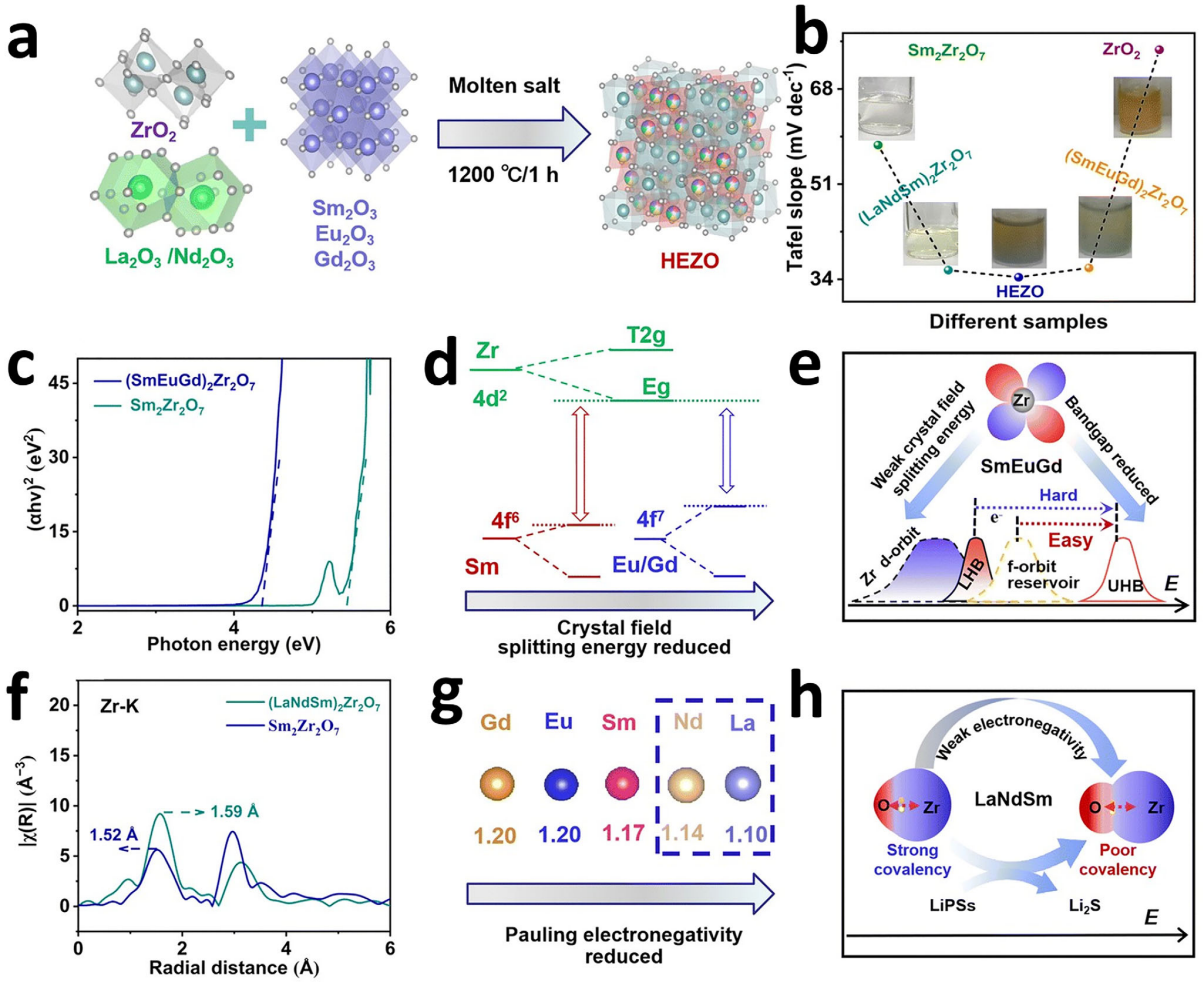
Descriptor‐driven regulation of lithium polysulfide adsorption through crystal‐field effects and electronegativity modulation in rare‐earth‐based high‐entropy pyrochlore oxides (HEZO). (a) Schematic of the molten salt synthesis of (La,Nd,Sm,Eu,Gd)_2_Zr_2_O_7_ from mixed rare‐earth oxides and ZrO_2_ at 1200°C for 1 h. (b) XRD patterns confirming a single‐phase pyrochlore structure. (c) Tauc plots derived from UV–vis spectroscopy, indicating bandgap changes due to high‐entropy engineering. (d) Schematic illustration of the reduction in crystal‐field splitting of Zr 4d orbitals (T_2_g–Eg gap) when Sm, Eu, and Gd substitute at the A‐site, weakening Zr–O covalency. (e) Comparison of Li_2_S_4_ adsorption behavior: weak on ZrO_2_, strong on Sm_2_Zr_2_O_7_, and optimally intermediate on HEZO. (f) Zr K‐edge EXAFS Fourier transform data showing the Zr–O bond distance increase from 1.52 Å (Sm_2_Zr_2_O_7_) to 1.59 Å ((LaNdSm)_2_Zr_2_O_7_), confirming structural modification. (g) Plot of decreasing Pauling electronegativity across the lanthanide series from La to Gd. (h) Summary mechanism: lower electronegativity of A‐site cations weakens Zr–O covalency, leading to moderate Li_2_S_4_ binding [27]. Adapted with permission from Ref. [27]. Copyright 2025, RSC.

Zhou et al. (2025) reported pyrochlore‐type high‐entropy oxides containing multiple REEs, demonstrating that rare‐earth site engineering can simultaneously modulate crystal‐field splitting and electronegativity, thereby optimising Li_2_S_4_ adsorption behavior [27]. As shown in Figure 2a, the molten salt synthesis of the high entropy pyrochlore (La,Nd,Sm,Eu,Gd)_2_Zr_2_O_7_ (HEZO) is achieved from mixed rare earth oxides and ZrO_2_ at 1200°C for 1 h. Figure 2b displays XRD patterns confirming a single‐phase pyrochlore structure without impurities, while Figure 2c presents Tauc plots revealing bandgap changes due to the incorporation of different lanthanides. The key mechanistic insight emerges from Figure 2d–h. Figure 2d illustrates how substituting Sm, Eu, and Gd at the A site reduces the crystal field splitting of the Zr 4d orbitals (the T_2_g–Eg gap), thereby weakening Zr–O covalency. Figure 2e compares Li_2_S_4_ adsorption on different surfaces: very weak on pure ZrO_2_, too strong on Sm_2_Zr_2_O_7_ (leading to possible passivation), and optimally intermediate on HEZO. Figure 2f provides Zr K‐edge EXAFS data, showing that the Zr─O bond distance increases from 1.52 Å in Sm_2_Zr_2_O_7_ to 1.59 Å in (LaNdSm)_2_Zr_2_O_7_, confirming that A‐site substitution alters the local structure. Figure 2g plots the decreasing Pauling electronegativity across the lanthanide series from La to Gd, which directly influences the ionicity of the Zr─O bond. Finally, Figure 2h summarizes the mechanism: reduced electronegativity of the A‐site cations weakens Zr–O covalency, leading to a moderate Li_2_S_4_ binding strength that is neither too strong nor too weak.

The broader principles of REE coordination chemistry have been systematically explored by Su et al. (2025), who demonstrated that tuning the local coordination environment can significantly alter electronic structure and catalytic properties [28]. While these insights provide a useful framework for engineering REE active sites, most current studies emphasize structural tunability without establishing direct correlations between coordination environment and sulfur redox kinetics, highlighting a persistent gap between material design and mechanistic validation. Beyond single‐atom and oxide‐based catalysts, REE‐containing compounds can also function as multifunctional sulfur hosts. For example, CeVO_4_ hollow spheres have been shown to simultaneously confine polysulfides and promote solid–liquid conversion kinetics, suggesting a coupled adsorption–conversion mechanism [29, 30]. Nevertheless, it remains unclear whether such behavior arises from intrinsic REE electronic effects. It could also arise from the composite structural features of the host material.

Taken together, current studies indicate that REE‐based catalysts operate through a combination of redox mediation, defect‐assisted electronic modulation, and polar adsorption. However, these mechanisms are often inferred indirectly, and their relative contributions remain poorly resolved. In particular, distinguishing true catalytic activity from strong adsorption effects remains a central challenge. Future research should therefore prioritize operando characterization techniques and quantitative kinetic analysis to establish definitive structure–function relationships in REE‐mediated sulfur conversion.

### 2.2 Ionic vs. Electronic Modulation at the 3D Interface

The cathode–electrolyte interface in Li–S batteries is governed by the coupled transport of electrons and Li^+^ ions. It also involves surface‐mediated sulfur conversion reactions. Effective catalyst design therefore requires simultaneous regulation of electronic conductivity, ionic accessibility, and interfacial reaction kinetics. However, most rare earth element (REE) compounds are intrinsically insulating, necessitating integration with conductive matrices to ensure efficient charge transport. For example, REE‐based single‐atom catalysts embedded within nitrogen‐doped carbon frameworks enable three‐dimensional conductive pathways, ensuring electronic accessibility to catalytically active sites. At the same time, REE ions act as strong Lewis acids with highly polar surfaces, enabling effective adsorption of negatively charged polysulfide species (S_x_^2^^−^). While such adsorption is beneficial for suppressing polysulfide shuttling, excessive binding can hinder desorption and slow down subsequent conversion reactions, leading to interfacial passivation. This highlights a critical trade‐off: optimal performance requires a balance between adsorption strength and catalytic turnover. The importance of interfacial electrostatics in regulating REE–polysulfide interactions has been demonstrated through theoretical studies. For instance, LaCount et al. showed via density functional theory (DFT) that local electric potentials can significantly modulate adsorption energetics. Substituent‐induced changes in oxygen Lewis basicity alter binding energies by 20–30 kcal mol^−1^ [31]. These results suggest that electrostatic tuning, rather than simply increasing polarity, is a key parameter in controlling interfacial reactivity.

In addition to adsorption effects, REE materials can influence ionic transport and interfacial conversion processes. For example, layered rare‐earth hydroxide/graphene oxide interlayers by Wang et al., 2024 have been shown to facilitate liquid–solid conversion while suppressing polysulfide migration, indicating that REEs can simultaneously regulate transport and reaction kinetics at the mesoscale [32]. However, such improvements are often attributed to composite structural effects, making it difficult to isolate the intrinsic contribution of REE chemistry. More fundamentally, the identity of the rare earth element plays a decisive role in determining interfacial electronic behavior. Li et al. (2021) demonstrated in amorphous ReAlO_3_/SrTiO_3_ heterointerfaces (Re = La, Pr, Nd, Sm, Gd, Tm) that the ionization potential of the REE cation acts as a key descriptor governing electronic transport properties, including sheet resistance and photoresponse [33].

**FIGURE 3:**
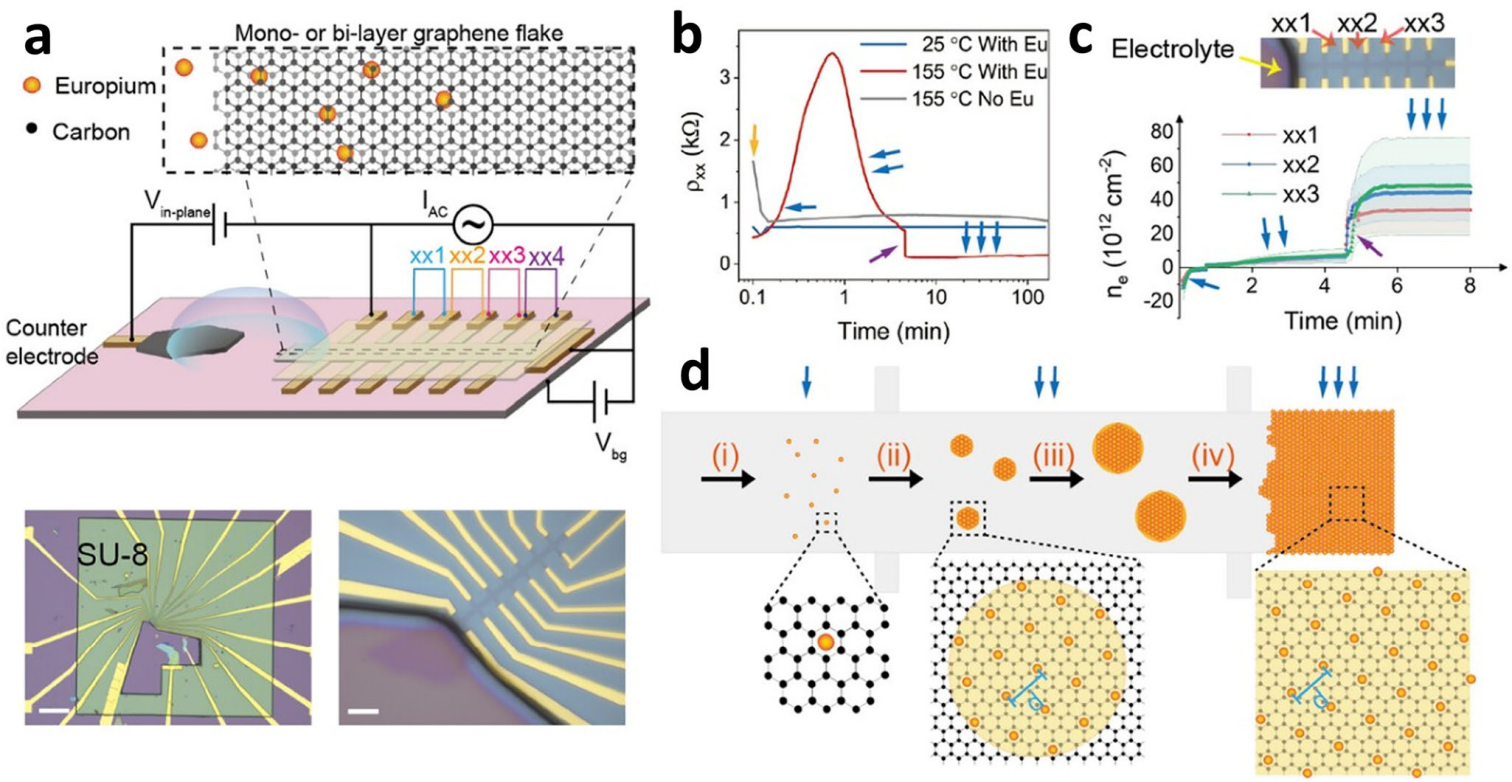
Reversible intercalation of europium ions into bilayer graphene and its effect on electronic transport properties. (a) Schematic of the device architecture. (b) Oxide resistance vs. time at different temperatures and with/without Eu. (c) Electron density from Hall measurements showing stepwise intercalation stages. (d) Optical microscopy images before and after intercalation confirming structural integrity. The results demonstrate that heavy lanthanides can form ordered two‐dimensional layers within bilayer graphene, dynamically modulating electronic properties at carbon interfaces a phenomenon with direct implications for interfacial regulation in Li–S batteries [34]. Adapted with permission from Ref. [34]. Copyright 2025, Wiley.

Notably, heavier lanthanides induce qualitatively different electronic phenomena, such as resistance upturns associated with weak localization, suggesting that REE selection can fundamentally alter interfacial electronic states rather than merely tuning adsorption strength. The interaction between REE ions and conductive carbon substrates has also been directly probed. Feng et al. (2025) reported reversible intercalation of europium ions into bilayer graphene, leading to the formation of ordered two‐dimensional Eu layers within the carbon framework [34]. To elucidate this process, detailed experimental characterization is presented in Figure 3. As depicted in Figure 3a, the device architecture consists of a bilayer graphene flake suspended over a trenched substrate with a counter electrode, while a back‐gate voltage is applied during exposure to a Eu‐containing electrolyte. Figure 3b plots the oxide resistance over time under three conditions: at 25°C with Eu, at 155°C with Eu, and at 155°C without Eu. Only the curve at 155°C with Eu shows a sharp drop shortly after bias application, demonstrating that Eu intercalation is both thermally activated and Eu‐dependent. Figure 3c presents the electron density extracted from Hall measurements, revealing a stepwise increase with distinct stages (xx1, xx2, xx3), providing clear evidence of ordered, layer‐by‐layer insertion of Eu atoms. Optical microscopy images confirming that the bilayer graphene maintains its structural integrity before and after intercalation are shown in Figure 3d.

### 2.3 DFT and Spectroscopic Insights on REE–Polysulfide Interactions

Mechanistic understanding of rare earth element (REE)–polysulfide (LiPS) interactions relies increasingly on combined methods. These include density functional theory (DFT) and advanced spectroscopic techniques. For Ce‐based systems, DFT calculations consistently show that lithium polysulfides preferentially bind to Ce‐centered active sites through coordination interactions, while defect engineering, particularly oxygen vacancy formation modulates adsorption strength and reaction energetics by introducing new hybridized electronic states. Extending beyond single‐component systems, recent studies on high‐entropy pyrochlore oxides demonstrate that descriptor‐level tuning, including crystal‐field splitting and electronegativity variation, can systematically regulate Li_2_S_4_ adsorption and conversion energetics, providing a quantitative framework for catalyst design [27].

Although many mechanistic insights originate from studies outside Li–S batteries, they offer valuable methodological guidance. For example, Sutton et al. (2020) employed periodic DFT calculations combined with vibrational sum‐frequency spectroscopy, ATR‐FTIR, and isothermal titration calorimetry. They elucidated ligand binding on Ce‐based mineral surfaces. Their work highlights the critical roles of coordination environment, solvent interactions, and surface electrostatics in governing adsorption behavior [35]. Such integrated theoretical–experimental approaches provide a useful blueprint for investigating REE–polysulfide interactions in battery systems. The reliability of DFT predictions for REE systems depends strongly on computational methodology. Bertoli et al. (2024) systematically evaluated functional selection and solvation effects across the lanthanide series, showing that hydrolysis reactions can be modeled with reasonable accuracy (≈5 kcal mol^−1^ error), whereas sulfate complexation exhibits larger uncertainties and sensitivity to functional choice [36]. Importantly, the agreement between calculated and experimental Raman spectra validates the use of vibrational signatures as a bridge between theory and experiment, reinforcing the importance of combining DFT with spectroscopic validation.

Experimentally, in situ and operando spectroscopic techniques have become essential for tracking sulfur redox chemistry. In situ Raman spectroscopy enables real‐time monitoring of LiPS evolution and phase transitions during cycling, as demonstrated in recent high‐entropy REE systems, where temporal mapping of S_8_ and Li_2_S_4_ signatures provides direct evidence of conversion pathways. Complementary surface‐sensitive techniques, including X‐ray photoelectron spectroscopy (XPS) and X‐ray absorption spectroscopy (XAS), further confirm that REE active sites undergo dynamic valence and coordination changes during operation, supporting their role as active catalytic centers rather than passive anchors. Synchrotron‐based techniques provide deeper insight into REE coordination environments. For instance, Zhao et al. (2025) combined in situ extended X‐ray absorption fine structure (EXAFS) spectroscopy with ab initio molecular dynamics (AIMD) simulations to resolve the temperature‐dependent coordination evolution of Yb^3+^ species [37]. Their results revealed a transition from hydrated coordination structures to sulfate‐dominated complexes, illustrating how REE coordination environments dynamically respond to chemical conditions. This integrated experimental–theoretical framework offers a powerful strategy for probing REE–polysulfide interactions in Li–S batteries.

**FIGURE 4:**
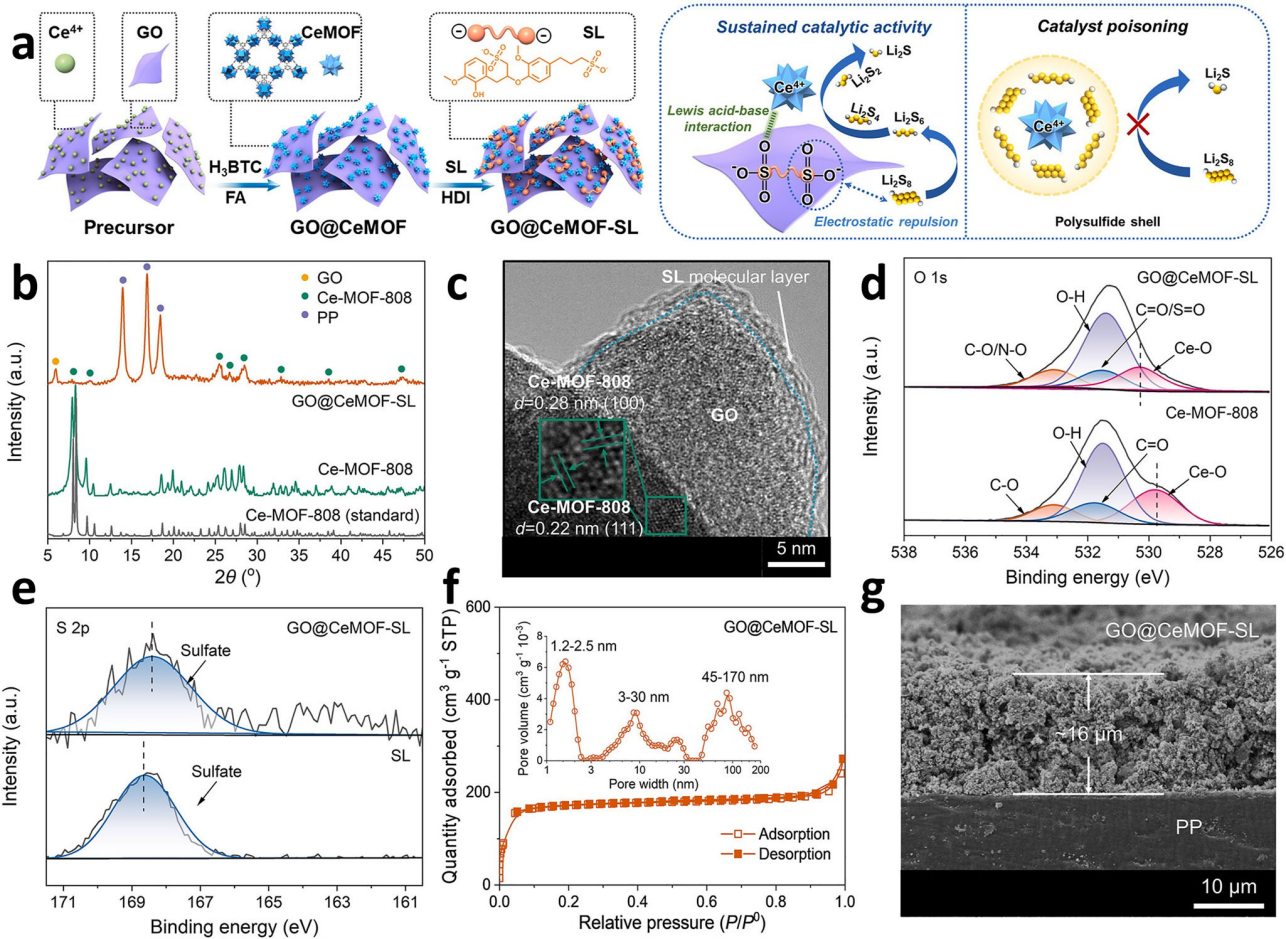
Electrostatic regulation of catalytic activity in REE‐based systems using GO@CeMOF‐SL. (a) Schematic synthesis of GO@CeMOF‐SL. (b) XRD patterns of GO, Ce‐MOF‐808, and GO@CeMOF‐SL confirming structural integrity. (c) HRTEM images showing Ce‐MOF‐808 lattice fringes (0.28 and 0.22 nm) and a ∼5 nm SL layer. (d) XPS O 1s spectra with additional C─O/N─O and S═O peaks after SL grafting. (e) XPS S 2p spectrum showing sulfate species from SL. (f) N_2_ adsorption–desorption isotherms and pore size distribution revealing hierarchical micro‐ and mesopores. (g) SEM image and EDS elemental maps showing uniform Ce, O, and S distribution. The electrostatic environment created by the SL layer stabilizes Ce^4+^ active sites, suppresses catalyst poisoning, and enables sustained sulfur redox activity [38]. Adapted with permission from Ref. [38]. Copyright 2026, Wiley.

In addition to understanding active sites, recent studies have emphasized the importance of controlling local chemical environments to prevent catalyst deactivation. For example, Zhang et al. (2026) demonstrated that introducing lignosulfonate (SL) into Ce‐MOF‐808 creates an electrostatic environment that suppresses catalyst poisoning while maintaining catalytic activity [38]. The synthesis of the GO@CeMOF‐SL composite is illustrated in Figure 4a, where Ce‐MOF‐808 is grown on graphene oxide and subsequently coated with lignosulfonate to form a hierarchical conductive network. Figure 4b shows the X‐ray Diffraction (XRD) patterns of graphene oxide (GO), Ce‐MOF‐808, and GO@CeMOF‐SL; the preserved Ce‐MOF‐808 peaks confirm that the crystal structure remains intact after SL incorporation. highy‐resolution transmission electron microscopy HRTEM images in Figure 4c reveal lattice fringes of Ce‐MOF‐808 with d‐spacings of 0.28 nm (100) and 0.22 nm (111), and a thin (∼5 nm) amorphous SL layer on the MOF surface. Figure 4d presents the XPS O 1s spectra: compared to Ce‐MOF‐808, GO@CeMOF‐SL shows additional C─O/N─O and S═O peaks, confirming successful SL grafting. Figure 4e displays the S 2p spectrum of GO@CeMOF‐SL, with a sulfate peak at ∼168 eV, further evidence of SL incorporation. Figure 4f presents the N_2_ adsorption–desorption isotherms and pore size distribution, revealing a hierarchical porosity with micropores (1.2–2.5 nm) from the MOF and mesopores (3–30 nm) from GO, which enhances electrolyte penetration and polysulfide accessibility. Finally, Figure 4g shows an scaning electron micrscopy (SEM) image andenergy dispersive x‐ray spectroscopy(EDS) elemental maps, confirming the uniform distribution of Ce, O, and S across the composite.

Importantly, the insights derived from density functional theory (DFT) and spectroscopic studies are increasingly reflected in practical electrochemical performance. As summarized in Table 1, a wide range of REE‐enabled systems, including single‐atom catalysts, vacancy‐engineered oxides, heterostructures, and interfacial modifiers, exhibit improved sulfur utilization, enhanced rate capability, and long‐term cycling stability under diverse operating conditions. However, the reported performance metrics vary widely in terms of sulfur loading, electrolyte conditions, and cell configuration, making direct comparison challenging. This underscores the need for standardized evaluation protocols and highlights the importance of linking mechanistic understanding with practically relevant performance metrics. Although DFT and spectroscopic approaches have provided valuable insights, current understanding remains fragmented. There is limited direct correlation between calculated descriptors and experimentally observed behavior. This disconnect highlights the need for more rigorous, quantitatively consistent approaches that combine real‐time characterization with theory to uncover the true catalytic mechanisms of REE‐based systems.

**TABLE 1:** Representative rare‐earth‐enabled interfacial catalytic systems for Li–S batteries and their reported electrochemical performance.

System (REE strategy)	Cell component	Sulfur loading (mg cm^−2^)	Representative capacity/rate	Cycling performance	Refs.
Ce–N coordination single‐atom sites in 3D conductive framework (Ce^3+^/Ce^4+^ mediation)	Cathode catalyst/host	∼1.5	1225 mAh g^−1^ @ 0.2C	76.1% retention after 160 cycles; pouch cell: 877 mAh g^−1^ after 50 cycles @ 0.5C	[39]
Vacancy‐engineered CeO_2_−_x_(Ce^3+^/Ce^4+^ + vacancy‐enhanced catalysis)	Cathode catalyst	—	—	743.2 mAh g^−1^ after 1000 cycles @ 0.5C	[40]
Lu single‐atom catalyst (Lu SA/NC) (f–d–p hybridization)	Cathode catalyst	5.96	1391.8 mAh g^−1^ @ 0.1C	0.049% capacity fading per cycle over 1000 cycles @ 1C	[41]
Layered rare‐earth hydroxides@GO interlayer (LGdH@GO; rare‐earth hydroxide + GO)	Functional interlayer/separator	—	605.34 mAh g^−1^ @ 5C	0.087% decay per cycle over 500 cycles @ 2C	[32]
MOF‐derived Pr_2_O_2_S–doped porous carbon nanosheets (Pr_2_O_2_S–C) separator coating	Separator coating	—	893 mAh g^−1^ @ 0.2C (after 200 cycles)	63% retention after 200 cycles @ 0.2C; 99.3% Coulombic efficiency	[42]
La^3+^‐doped Sn_3_O_4_ nanoflowers (4f electron configuration catalysis)	Cathode host	—	1429.6 mAh g^−1^ @ 0.1C	76.5% retention after 130 cycles @ 0.1C	[43]
Nd_2_O_2_S‐doped porous carbon nanocones (M‐O/M‐S dual‐anionic centers)	Separator coating	—	902 mAh g^−1^ @ 0.2C (after 1000 cycles)	77.5% retention after 1000 cycles @ 0.2C; 99.3% Coulombic efficiency	[44]
Gd_2_O_3_/Co@NC heterojunction (TMs‐REOs coupling)	Separator modifier	5.1	628.0 mAh g^−1^ @ 4C	504.2 mAh g^−1^ after 500 cycles @ 2C; areal capacity 4.8 mAh cm^−2^	[45]
Eu‐incorporated NiSe (d‐p‐f orbital coupling)	Cathode catalyst	5.94	896.2 mAh g^−1^ @ 4C	657.3 mAh g^−1^ after 500 cycles @ 1C; areal capacity 5.66 mAh cm^−2^	[46]
Lu_2_O_3_ (f‐d hybridization descriptor optimization)	Solid‐state cathode catalyst	—	—	>20,000 cycles @ 5C; areal capacity 14.48 mAh cm^−2^ (all‐solid‐state)	[47]

### 2.4 Distinguishing Adsorption From Catalysis: A Proposed Methodology

A persistent challenge in REE‐based Li–S catalysts is disentangling genuine catalytic activity (acceleration of redox conversion) from mere adsorption (physical/chemical retention). Strong adsorption is often mistaken for high catalytic efficiency, but excessive binding can poison active sites and hinder desorption. This section proposes a three‐pillar methodology: theoretical pre‐screening, electrochemical decoupling, and operando spectroscopy to distinguish these two effects. The first pillar is DFT pre‐screening based on the “optimal adsorption” principle. Optimal Li–S performance follows a three‐step chain of adsorption, catalysis, and desorption, and excessively strong adsorption disrupts the later steps. Across diverse catalyst systems, a volcano trend appears: neither the weakest nor the strongest adsorption yields the best performance; instead, moderate adsorption is optimal [48]. Because REEs lack a simple d‐band descriptor, DFT calculations must evaluate not only the adsorption energy (ΔG) of key intermediates (Li_2_S_4_, Li_2_S) but also the Li_2_S nucleation barrier, oxygen vacancy formation energy, and f–d hybridization (when REEs are combined with transition metals) [49]. A material with extremely negative ΔG but no reduction in the Li_2_S nucleation barrier is adsorption‐dominated, whereas one with moderate ΔG and a significantly lowered barrier qualifies as a true catalyst. Machine learning and high‐throughput screening can accelerate this pre‐screening process [50]. The second pillar is electrochemical decoupling, using kinetics as the fingerprint. A mere adsorbent does not alter the reaction pathway, whereas a true catalyst lowers the Tafel slope and activation energy. The most direct probe is the Li_2_S nucleation overpotential: catalysis‐dominated behavior gives a low overpotential (< 50 mV), while adsorption‐dominated behavior gives a high overpotential (> 80 mV) [51]. Measuring this overpotential against non‐catalytic controls provides a quantitative metric.

The third pillar is operando spectroscopy to visualize the dynamic process. Operando techniques directly observe whether REE sites actively participate in electron transfer or merely provide a polar surface. Raman microscopy tracks intermediate lifetimes: short lifetimes indicate rapid conversion (catalysis), whereas long lifetimes indicate accumulation (adsorption) [52]. XAS monitors reversible redox changes (e.g., Ce^3+^/Ce^4+^, Eu^2+^/Eu^3+^) and the formation/breaking of REE–S bonds. A static oxidation state indicates adsorption; reversible cycling proves catalytic mediation [40]. Table 2 summarizes the quantitative thresholds for each pillar.

**TABLE 2:** Multi‐parameter evaluation matrix for distinguishing adsorption‐dominated from catalysis‐dominated behavior in REE‐based Li–S catalysts.

Metric	Adsorption‐dominated	Catalysis‐dominated	Refs.
Li_2_S_4_ adsorption energy (DFT)	Highly negative (< −3.5 eV)	Moderate (−1.5 to −3.0 eV)	[53]
Tafel slope	Unchanged or slightly reduced	Significantly reduced	[54]
Li_2_S nucleation overpotential	High (> 80 mV)	Low (< 50 mV)	[51]
Activation energy (E_a_)	Similar to baseline	Markedly lower	[55]
Operando Raman (intermediate lifetime)	Long (accumulation)	Short (rapid conversion)	[52]
Operando XAS (REE redox)	Static	Reversible cycling	[40]

The takeaway is clear: a REE material is a true catalyst only if all three pillars are satisfied. That is, it must show moderate DFT adsorption energy (−1.5 to −3.0 eV) together with a lowered Li_2_S nucleation barrier; it must exhibit a low Li_2_S overpotential (< 50 mV) and a reduced Tafel slope in electrochemical tests; and it must demonstrate reversible redox cycling by operando XAS and short intermediate lifetimes by operando Raman. If a material scores well on adsorption capacity but fails on kinetic or operando metrics, it should be classified as an adsorbent, not a catalyst.

Despite growing computational and spectroscopic evidence, several critical gaps remain. No systematic DFT comparison across the full lanthanide series exists; a contradiction persists between DFT (which often suggests that stronger adsorption always benefits confinement) and experiment (which shows a volcano trend); operando XAS studies on REE redox dynamics are scarce; and nearly all computational studies neglect solvation and electrolyte effects, which can alter adsorption energies by more than 1 eV. Addressing these gaps requires coordinated, multi‐laboratory efforts using standardized protocols.

## 3 Rare‐Earth Composite Materials for Li–S Systems

The integration of REEs into diverse material platforms has enabled the development of multifunctional composites. These composites address key limitations in lithium–sulfur (Li–S) batteries, including the polysulfide shuttle effect, sluggish redox kinetics, and structural instability. Rather than acting as standalone catalysts, REEs are typically incorporated into composite architectures such as carbon matrices, metal–organic frameworks (MOFs), oxides, chalcogenides, and hybrid systems where their chemical functionality complements the physical and electrical properties of the host material.

Recent advances highlight that the role of REEs extends beyond traditional catalyst design toward multifunctional integration at multiple length scales. For example, REE incorporation into polymeric binders introduces catalytic functionality directly within the electrode matrix. Zhou et al. (2024) developed an aqueous supramolecular binder (GB–Y) based on a yttrium–pyridine complex, in which Y^3+^ sites functioned as Lewis acid centers to accelerate polysulfide conversion while hydrogen bonding maintained electrode integrity, resulting in high initial capacity (1201.5 mAh g^−1^) and stable cycling over 800 cycles [56]. This approach demonstrates that REEs can be embedded into non‐traditional components, expanding their functional role beyond conventional catalyst or host materials. Similarly, heterostructured systems incorporating multiple REEs have shown enhanced catalytic activity through interfacial synergy.

Rare‐earth oxysulfides represent another emerging class of functional materials with balanced adsorption–conversion properties. Sun et al. (2023) demonstrated that La_2_O_2_S exhibits moderate adsorption energy and favorable reaction thermodynamics compared to La_2_O_3_ and La_2_S_3_, enabling improved polysulfide regulation when used in separator modification [57]. This suggests that tuning anion composition (O vs. S) can provide an additional degree of freedom for optimizing REE‐based catalytic behavior. More recently, the development of rare‐earth single‐atom catalysts (SACs) has opened new directions for maximizing atomic utilization and exposing active sites. Zhou et al. (2024) reported a Sm‐based SAC system in which electron‐rich 4f orbitals, coupled with f–d–p hybridization, promoted polysulfide conversion and uniform lithium deposition [58]. Compared to conventional transition‐metal SACs, which often suffer from passivation, REE SACs may offer enhanced resistance to deactivation. Nevertheless, the precise role of 4f orbitals in catalytic activity remains insufficiently resolved, and direct experimental validation of proposed electronic mechanisms is still limited. These examples illustrate that REE‐based composites derive their performance not from a single function, but from the integration of catalytic activity, adsorption capability, and structural regulation within complex architectures. However, the diversity of material systems and testing conditions complicates direct comparison, underscoring the need for clearer structure–function correlations and standardized evaluation criteria.

### 3.1 Rare‐Earth Carbon Composites

**FIGURE 5:**
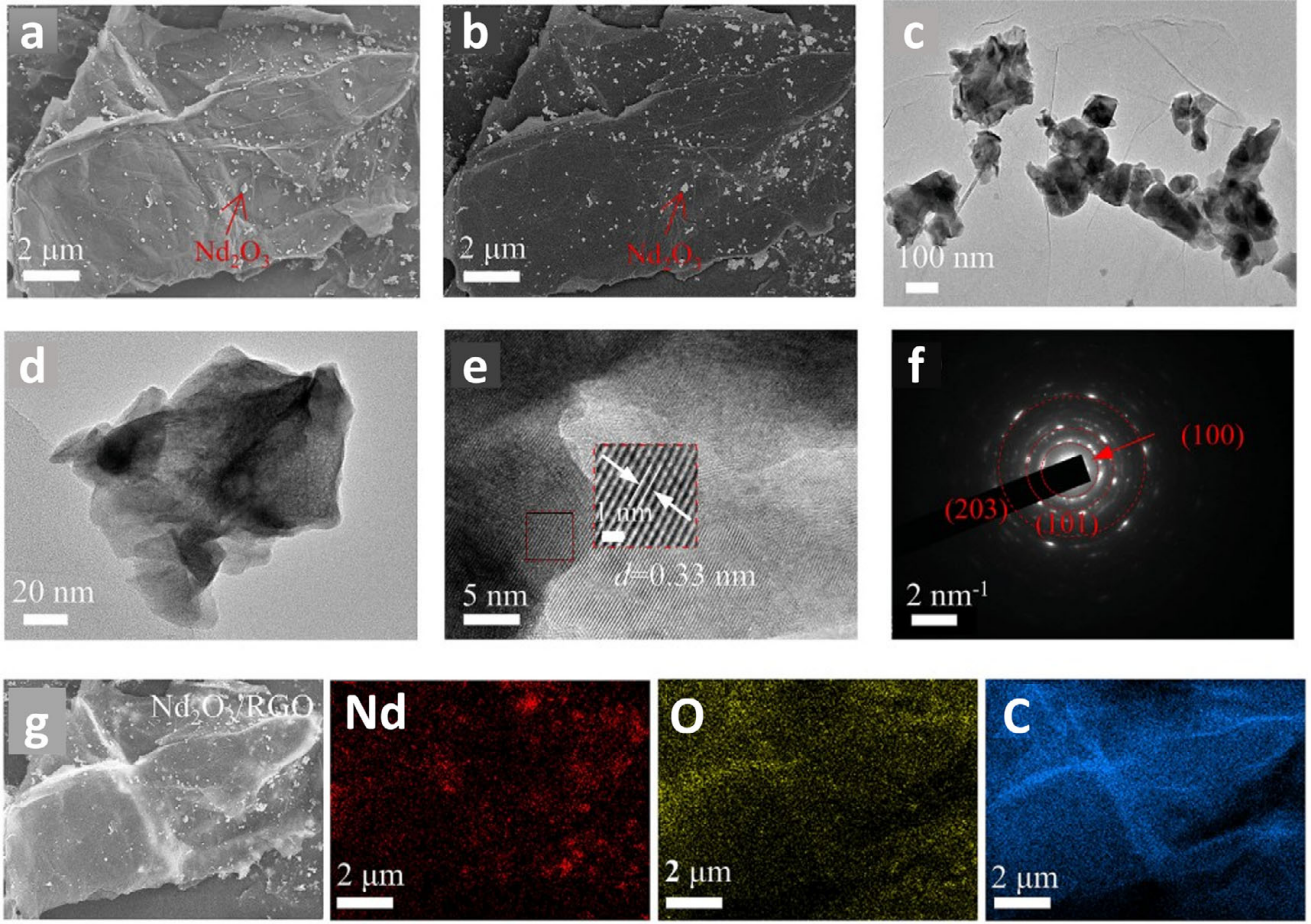
Structural integration of Nd_2_O_3_ nanoparticles with RGO to form a conductive and chemically active composite. (a) Low‐magnification SEM image showing uniform dispersion of Nd_2_O_3_ nanoparticles on wrinkled RGO sheets. (b) Higher‐magnification SEM image revealing spherical Nd_2_O_3_ particles (50–200 nm diameter) without aggregation. (c) TEM image confirming that Nd_2_O_3_ nanoparticles are embedded on and between RGO layers. (d) HRTEM image of a single Nd_2_O_3_ nanoparticle showing lattice fringes with a d‐spacing of ∼0.33 nm, corresponding to the (100) or (203) planes of hexagonal Nd_2_O_3_. (e) Higher‐magnification HRTEM image showing well‐ordered lattice fringes (d = 0.33 nm), confirming high crystallinity. (f–g) EDS elemental maps: Nd (red), O (green), C (blue). Adapted with permission [59]. Adapted with permission from Ref. [59]. Copyright 2024, Elsevier.

Carbon materials remain the most widely used platforms for Li–S cathodes due to their high electrical conductivity, tunable porosity, and mechanical robustness. The incorporation of REEs into carbon frameworks creates synergistic composites in which carbon provides efficient electron transport, while REE sites introduce strong chemical interactions with polysulfides and catalytic functionality. For example, Zheng et al. (2024) reported Nd_2_O_3_/reduced graphene oxide (RGO) composites for separator modification, where the Lewis acidic Nd_2_O_3_ sites interact strongly with polysulfide species, suppressing the shuttle effect while maintaining electronic conductivity [59]. The structural integration of Nd_2_O_3_ nanoparticles within conductive RGO sheets ensures both effective adsorption and rapid charge transport, highlighting the necessity of intimate contact between REE active sites and conductive networks to achieve efficient catalytic performance. Figure 5 provides direct microscopic evidence of this integrated architecture. Specifically, SEM Figure 5a–c reveals that Nd_2_O_3_ nanoparticles are uniformly dispersed across and within the wrinkled RGO sheets, forming a continuous and interconnected structure that maximizes exposure of active sites while maintaining electrical pathways. HRTEM images in Figure 5d,e confirm the crystalline nature of the nanoparticles, with well‐defined lattice fringes corresponding to hexagonal Nd_2_O_3_, indicative of structurally stable catalytic domains. EDS in Figure 5f,g further demonstrates the co‐localization of Nd and O signals, verifying the formation of Nd_2_O_3_, while the carbon signal (panel g) shows that the nanoparticles are embedded within an interconnected conductive network, ensuring efficient electronic contact.

These observations collectively establish a key structure–function relationship: uniform dispersion of REE nanoparticles within a conductive carbon framework enables simultaneous optimization of polysulfide adsorption and charge transport. This synergy is essential, as isolated REE particles would suffer from poor conductivity, whereas carbon alone lacks sufficient chemical anchoring capability. The system therefore exemplifies how rational structural integration, rather than individual material properties, governs catalytic effectiveness in Li–S batteries.

Furthermore, insights from related materials studies further clarify the role of REE–carbon interactions. For instance, surface functionalization strategies involving ligand coordination (e.g., PAN–REE chelation) demonstrate how REE ions can be uniformly dispersed and stabilized on carbon substrates. These approaches provide useful design principles for controlling active site distribution and preventing aggregation in Li–S cathodes. The effectiveness of REE–carbon composites fundamentally arises from their complementary functionalities. First, carbon frameworks provide continuous electron pathways, ensuring that otherwise insulating REE compounds remain electrochemically accessible. Second, dual confinement mechanisms emerge, where physical trapping within carbon pores is coupled with chemical anchoring at REE sites, enhancing polysulfide retention. Third, the porous and flexible structure of carbon materials accommodates the significant volume expansion associated with sulfur conversion, improving structural stability.

In addition to these roles, strong metal–support interactions can further modify the electronic structure of REE sites. Interactions with nitrogen‐doped carbon, for example, can induce orbital coupling and spin polarization effects, potentially enhancing catalytic activity [60, 61, 62]. Furthermore, carbon matrices enable the stabilization of atomically dispersed REE species through strong coordination, which is critical for maintaining high catalytic efficiency [63]. However, many reported REE–carbon systems incorporate additional functionalities such as improved graphitization, enhanced dielectric properties, or structural reinforcement that are not directly related to Li–S electrochemistry. While these studies provide valuable insights into material design, their direct relevance to sulfur redox chemistry should be interpreted cautiously. This highlights an important limitation: improvements in composite properties do not necessarily translate into enhanced catalytic performance unless explicitly linked to sulfur conversion mechanisms.

### 3.2 Rare‐Earth Metal–Organic Frameworks (REE‐MOFs)

Rare‐earth metal–organic frameworks (REE‐MOFs) provide a unique platform for Li–S batteries by combining atomically dispersed metal centers with well‐defined coordination environments and tunable pore architectures. These features enable simultaneous control over polysulfide adsorption, catalytic conversion, and mass transport, making REE‐MOFs fundamentally different from conventional bulk catalysts. In particular, the presence of unsaturated REE sites and polar metal–oxygen clusters introduces strong Lewis acid–base interactions with polysulfide species, while the porous framework offers size‐selective confinement. For example, Hong and co‐workers reported a Ce‐MOF/CNT composite as a separator coating, where polar Ce–O clusters provided strong polysulfide affinity and improved cycling stability, delivering 569.3 mAh g^−1^ at 2 C with 88% retention after 200 cycles [64]. In such systems, polysulfide regulation arises from a combination of mechanisms, including (i) Lewis acid–base coordination at unsaturated Ce^3+^/Ce^4+^ sites, (ii) physical confinement within the pore network, and (iii) catalytic promotion of polysulfide conversion. However, these contributions are often difficult to decouple, particularly when conductive additives such as carbon nanotubes are simultaneously present.

**FIGURE 6:**
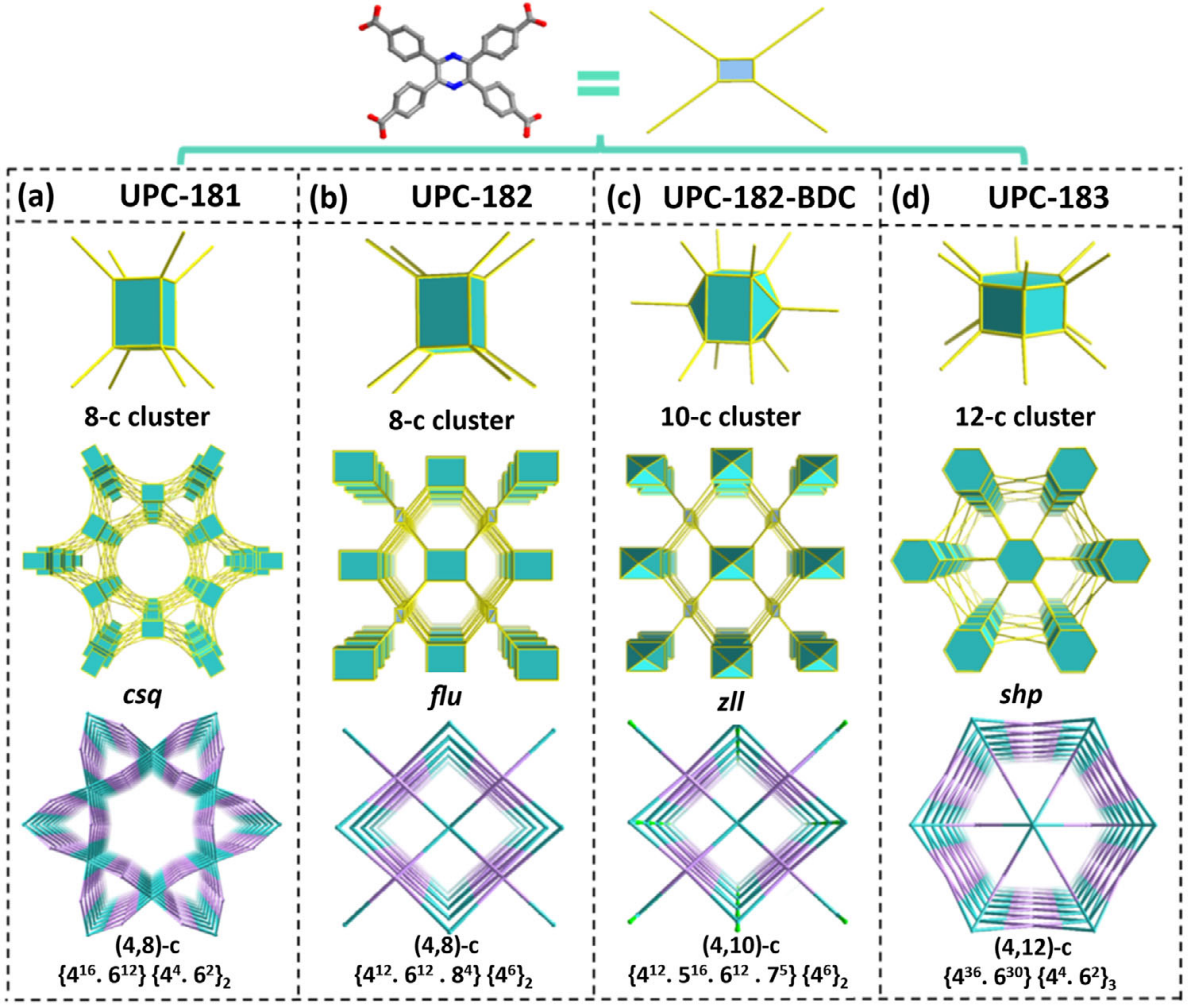
Structural diversity of REE‐MOF arising from variations in cluster nuclearity and linker symmetry. Four representative MOFs are shown (UPC‐181, UPC‐182, UPC‐182‐BDC, UPC‐183). For each, the figure displays: the metal cluster (green polyhedra) with its nuclearity (8‐c, 10‐c, or 12‐c); the organic linker (blue rods); the network topology (csq, flu, zll, or shp); and the 3D framework with pore channels. UPC‐181 (8‐c cluster, linear linker) → csq topology (square channels). UPC‐182 (8‐c cluster, bent linker) → flu topology (more open structure). UPC‐182‐BDC (10‐c cluster) → zll topology (elongated pores). UPC‐183 (12‐c cluster) → shp topology (hexagonal channels). This figure highlights how identical REE building units can generate multiple network topologies through modifications in linker geometry and coordination modes, demonstrating the tunability of pore architecture and coordination environments for designing polysulfide‐regulating materials. Adapted with permission [65]. Adapted with permission from Ref. [65]. Copyright 2022, Elsevier.

Beyond Li–S applications, studies on REE‐MOF stability and adsorption behavior provide important insights into structure–function relationships. Guo et al. (2022) demonstrated that nonanuclear, highly connected REE‐MOFs (e.g., UPC‐183‐Eu) exhibit superior stability and adsorption capacity compared to lower‐connectivity analogs, achieving a selenite uptake of 308.39 mg g^−1^ through combined chemisorption and physisorption [65]. While this system is not directly related to Li–S chemistry, it highlights the critical role of cluster nuclearity and connectivity in determining adsorption strength and framework robustness factors that are equally relevant for polysulfide confinement. The structural tunability of REE‐MOFs represents one of their most significant advantages. By varying linker geometry, symmetry, and coordination modes, it is possible to precisely control pore size, topology, and surface chemistry. Figure 6 illustrates this concept by comparing a series of REE‐MOFs (UPC‐181, UPC‐182, UPC‐182‐BDC, and UPC‐183) constructed from similar building units but exhibiting distinct network topologies.

Specifically, the figure shows how variations in cluster nuclearity (8 c, 10 c, and 12 c) and linker geometry lead to different framework architectures, including csq, flu, zll, and shp topologies, each with characteristic pore structures and channel geometries. These structural differences directly influence adsorption environments and mass transport pathways within the framework.

This comparison highlights a key design principle: framework topology in REE‐MOFs is not fixed but can be systematically tuned through coordination chemistry, enabling precise control over pore architecture and adsorption behavior. Such tunability is particularly valuable for Li–S batteries, where optimal performance requires a balance between strong polysulfide confinement and efficient ionic transport. Therefore, insights from these model systems provide a foundation for rationally designing REE‐MOF‐based hosts with tailored structure–property relationships.

Further control over framework properties can be achieved through linker engineering and multi‐metal incorporation. For instance, Lv et al. (2020) reported mesoporous REE‐MOFs constructed from *π*‐stacked pyrene‐based linkers and RE_6_ clusters, creating highly open structures with enhanced accessibility [66]. Similarly, Li et al. (2023) demonstrated that ortho‐functionalized tricarboxylate ligands can direct cluster formation, leading to the coexistence of hexanuclear and tetranuclear nodes [67]. More recently, multivariate REE‐MOFs incorporating multiple lanthanide ions (e.g., La, Ce, Eu, Gd, Tb, Dy, Y, Yb) have been developed, enabling compositional tuning of electronic and chemical properties within a single framework [68]. While such complexity offers opportunities for synergistic effects, it also introduces challenges in identifying the specific role of each REE component.

Morphological and post‐synthetic modifications further expand the functionality of REE‐MOFs. Controlled synthesis can yield diverse morphologies, including hexagonal‐bipyramidal, dendritic, and snowflake‐like structures, which can be transformed into derived materials (e.g., CeO_2_/Co/C hybrids) while preserving structural features [69]. Additionally, post‐synthetic modification strategies, such as linker exchange and metalation, enable the incorporation of multiple active sites within a single framework, as demonstrated in PCN‐900(RE) systems [70]. Coordination environment tuning has also been shown to influence performance; for example, six‐coordinated REE centers in CPM‐66 exhibited superior separation efficiency compared to seven‐coordinated analogs, underscoring the importance of local coordination geometry [71]. Coordination environment tuning has also been shown to influence performance; for example, six‐coordinated REE centers in CPM‐66 exhibited superior separation efficiency compared to seven‐coordinated analogs, underscoring the importance of local coordination geometry.

### 3.3 Rare‐Earth Oxides (REOs) and Mixed Metal Oxides

Rare‐earth oxides (REOs) represent the most extensively investigated class of REE‐based materials for Li–S batteries, primarily due to their strong polarity, tunable acid–base properties, and accessible redox chemistry. Unlike carbon‐based hosts that rely mainly on physical confinement, REOs provide chemically active surfaces capable of interacting strongly with lithium polysulfides (LiPSs), thereby enabling both adsorption and catalytic conversion. Their versatility also allows integration into a wide range of composite and heterostructured systems.

The fundamental properties of REOs are highly sensitive to composition, synthesis conditions, and lanthanide identity. Early systematic studies by Ivanova demonstrated that parameters such as phase composition, surface enrichment, pore structure, and acid–base characteristics vary significantly with REE type and concentration, directly influencing surface reactivity [72].

In particular, the acid–base nature of REOs plays a central role in determining their interaction with polysulfide species. Sato et al. showed that basic site strength follows the lanthanide contraction trend, decreasing with decreasing ionic radius, such that light REOs (e.g., La_2_O_3_, Pr_6_O_11_, Nd_2_O_3_, Sm_2_O_3_) exhibit strong basicity, while heavy REOs possess comparatively weaker basic sites [73]. This implies that light lanthanides may favor stronger polysulfide adsorption, whereas heavier lanthanides may offer weaker but potentially more reversible interactions. Surface chemistry further contributes to REO functionality. Lundy et al. demonstrated that REOs are intrinsically hydrophilic, with high surface energies and strong affinity for polar species, indicating that their interaction with LiPSs is fundamentally driven by surface polarity rather than incidental surface contamination [74]. In the context of Li–S batteries, such hydrophilicity is advantageous for anchoring soluble polysulfides but may also influence electrolyte wetting and interfacial stability.

Among REOs, CeO_2_ has received the most attention due to its ability to form oxygen vacancies. These features enable dynamic modulation of electronic structure and catalytic activity. For example, CeO_2_ incorporated into MOF‐derived porous carbon frameworks has been shown to exhibit strong Ce–S interactions, enhancing polysulfide adsorption and promoting conversion kinetics [75]. Importantly, the catalytic behavior of CeO_2_ is often attributed not only to its redox activity but also to defect‐mediated electronic states, which can facilitate charge transfer and intermediate stabilization.

However, single‐component REOs often face limitations, particularly their low intrinsic electronic conductivity and the trade‐off between strong adsorption and catalytic turnover. To address these challenges, heterostructure engineering has emerged as an effective strategy. Dooley et al. demonstrated that incorporating transition metal oxides such as MnO_x_ or FeO_x_ into REO systems significantly enhances sulfur capture capacity compared to single oxides, indicating that cooperative redox and adsorption mechanisms can improve overall performance [76]. Although these systems operate under different conditions, the underlying principle of synergistic interaction between REEs and transition metals is directly relevant to Li–S catalysis.

Recent advances in multicomponent and high‐entropy oxide systems have introduced additional complexity and tunability. Sarkar et al. showed that entropy‐stabilized oxides containing multiple cations can form phase‐pure structures with enhanced structural stability compared to lower‐entropy systems [77]. Similarly, Djenadic et al. demonstrated that cerium plays a key role in stabilizing multicomponent oxide phases into single‐phase fluorite structures [78]. These findings suggest that compositional complexity can be leveraged to tune electronic structure, defect density, and catalytic behavior. However, increasing compositional diversity also complicates mechanistic interpretation, as the individual contributions of each component become difficult to isolate.

Overall, REOs and mixed metal oxides provide a versatile platform for regulating polysulfide adsorption and conversion through a combination of surface polarity, redox activity, and defect chemistry. Nevertheless, several challenges remain. The intrinsic insulating nature of most REOs necessitates integration with conductive matrices, while strong adsorption may lead to sluggish desorption and reduced reversibility if not properly balanced. Moreover, the relationship between lanthanide identity, electronic structure, and catalytic performance is still not fully understood, particularly for mid‐ and heavy‐lanthanides. A key direction for future research is therefore the development of descriptor‐based design strategies that link acid–base properties, defect chemistry, and electronic structure to sulfur redox kinetics. In particular, systematic comparisons across the lanthanide series under identical conditions are needed to establish clear structure–property–performance relationships and to move beyond the current reliance on a limited set of commonly studied elements such as cerium.

### 3.4 Design Principles for REE‐Based Composite Materials

The design of REE‐based composite materials for Li–S batteries requires a holistic consideration of electronic conductivity, interfacial chemistry, and catalytic functionality. A central requirement is conductive integration, as most REE compounds are intrinsically insulating and must be intimately coupled with conductive matrices to ensure electrochemical accessibility of active sites. Fundamental insights into REE surface behavior provide important guidance for material design. For instance, Carchini et al. demonstrated that the surface wetting of REOs is governed by both geometric factors, such as lattice matching between adsorption sites and molecular networks, and electronic factors related to surface acid–base properties [79]. Similarly, Gupta et al. showed that while transition metal substitution in ceria significantly enhances reducibility through distortion of cation–oxygen bonds, substitution with other rare‐earth ions produces comparatively limited effects, indicating that not all REE modifications lead to meaningful improvements in catalytic functionality [80]. These findings highlight that structural modification alone is insufficient unless it directly alters electronic structure and reactivity.

**FIGURE 7:**
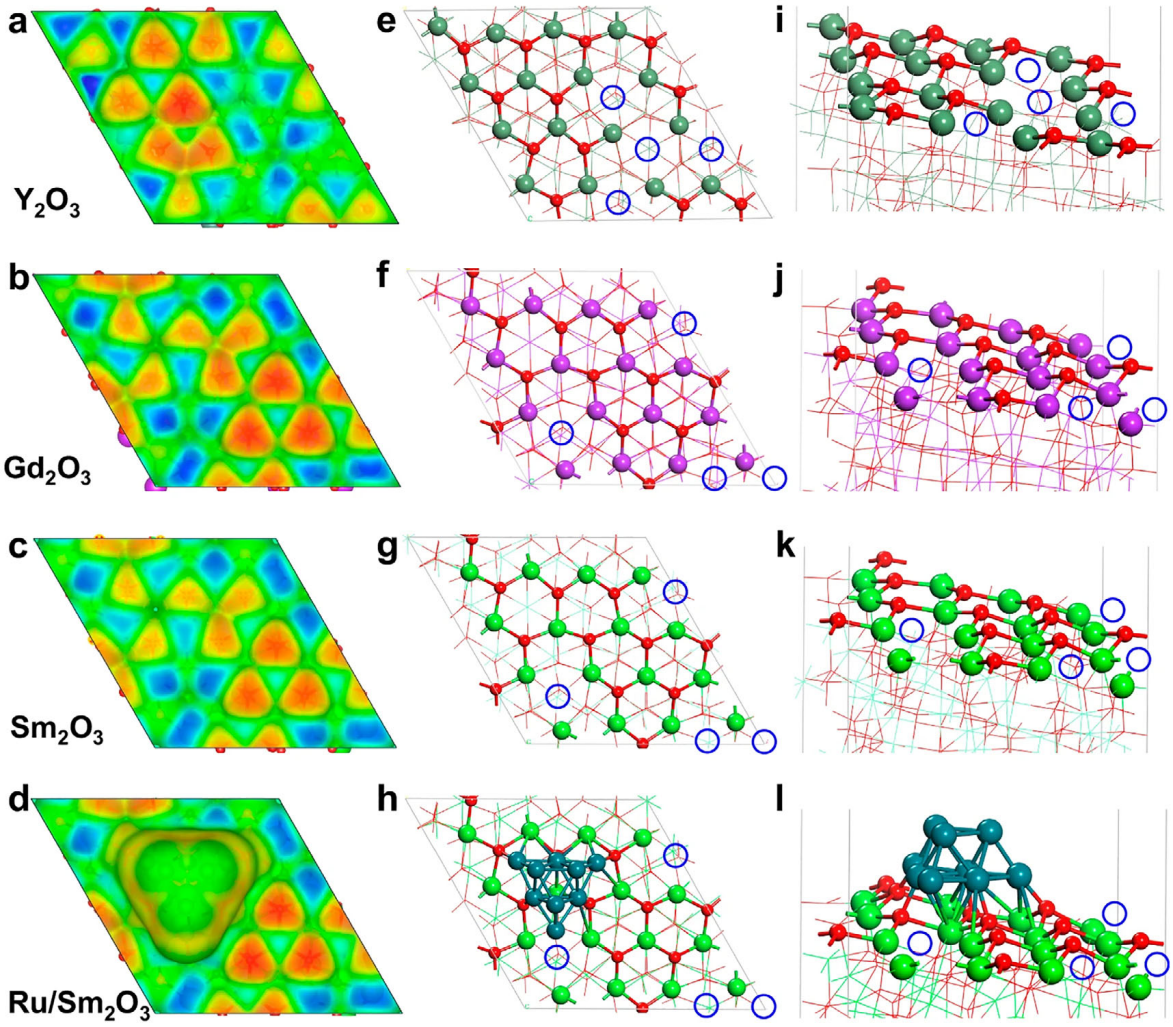
Defect‐induced electronic modulation on RE_2_O_3_(111) surfaces revealed by DFT calculations. (a–d) Electrostatic potential maps of Y_2_O_3_, Gd_2_O_3_, Sm_2_O_3_, and Ru‐modified Sm_2_O_3_ surfaces, showing charge polarization with electrophilic (blue) and nucleophilic (orange) regions associated with intrinsic oxygen vacancies. (e–h) Top views of optimized surface structures, illustrating the hexagonal sawtooth reconstruction composed of 5‐coordinated RE atoms and 4‐coordinated oxygen atoms. (i–l) Side views of the corresponding surfaces, highlighting the corrugated morphology and spatial distribution of vacancy sites. Blue circles indicate intrinsic oxygen vacancies [81]. Adapted with permission from Ref. [81]. Copyright 2024, Springer Nature.

Defect engineering represents another critical design strategy. Xu et al. (2024) revealed that intrinsic oxygen vacancies can arise from the irregular hexagonal sawtooth structure of RE_2_O_3_(111) surfaces, generating highly active sites for catalytic processes [81]. Figure 7 illustrates this behavior through DFT analysis of Y_2_O_3_, Gd_2_O_3_, and Sm_2_O_3_ (111) surfaces, along with a Ru‐modified Sm_2_O_3_ system. The electrostatic potential maps are represented in Figure 7a for Y_2_O_3_, Figure 7b for Gd_2_O_3_, Figure 7c for Sm_2_O_3_, and Figure 7d for Ru‐modified Sm_2_O_3_, which means blue regions correspond to electrophilic sites (electron‐loving) and orange regions to nucleophilic sites (electron‐rich). This charge polarization arises from intrinsic oxygen vacancies formed when surface oxygen atoms are removed, exposing undercoordinated rare‐earth cations.

The optimized atomic structures are shown in top‐view Figure 7e–h, which means the hexagonal sawtooth reconstruction of the RE_2_O_3_(111) surface is visible, featuring alternating 5‐coordinated RE atoms and 4‐coordinated oxygen atoms. This geometry inherently creates a high density of intrinsic oxygen vacancies (∼25% of surface oxygen sites). Side‐view structures Figure 7i–l highlight the corrugated surface topology and spatial distribution of vacancy sites, confirming their structural stability. In the Ru‐modified Sm_2_O_3_ system, the electrostatic potential map Figure 7d, the top‐view atomic structure Figure 7h, and the side‐view structure Figure 7l collectively show that the introduction of Ru clusters further modifies the local electronic environment. This modification enhances adsorption strength enough to activate reactants while avoiding excessive binding that could lead to catalyst poisoning.

Computational screening further enables rational selection of REE components. Wang et al. (2022) demonstrated that heavy lanthanides can outperform light ones in certain oxide systems, with Yb exhibiting lower formation energy and enhanced transition‐metal–oxygen hybridization, leading to improved electrochemical performance [82]. This suggests that lanthanide selection should be guided by electronic descriptors, rather than empirical preference for commonly studied elements such as cerium or lanthanum. Electronic structure engineering can also be achieved through spin‐state modulation. Li et al. (2025) showed that incorporation of Sm^3+^, Nd^3+^, and Ho^3+^ into MnCo_2_O_4_._5_ enhances Co–O covalency and induces 4f–3d coupling, driving the system toward mixed‐spin configurations that lower adsorption energy barriers and improve catalytic kinetics [83]. These results indicate that REEs can influence not only adsorption strength but also the electronic pathways governing reaction kinetics.

**FIGURE 8:**
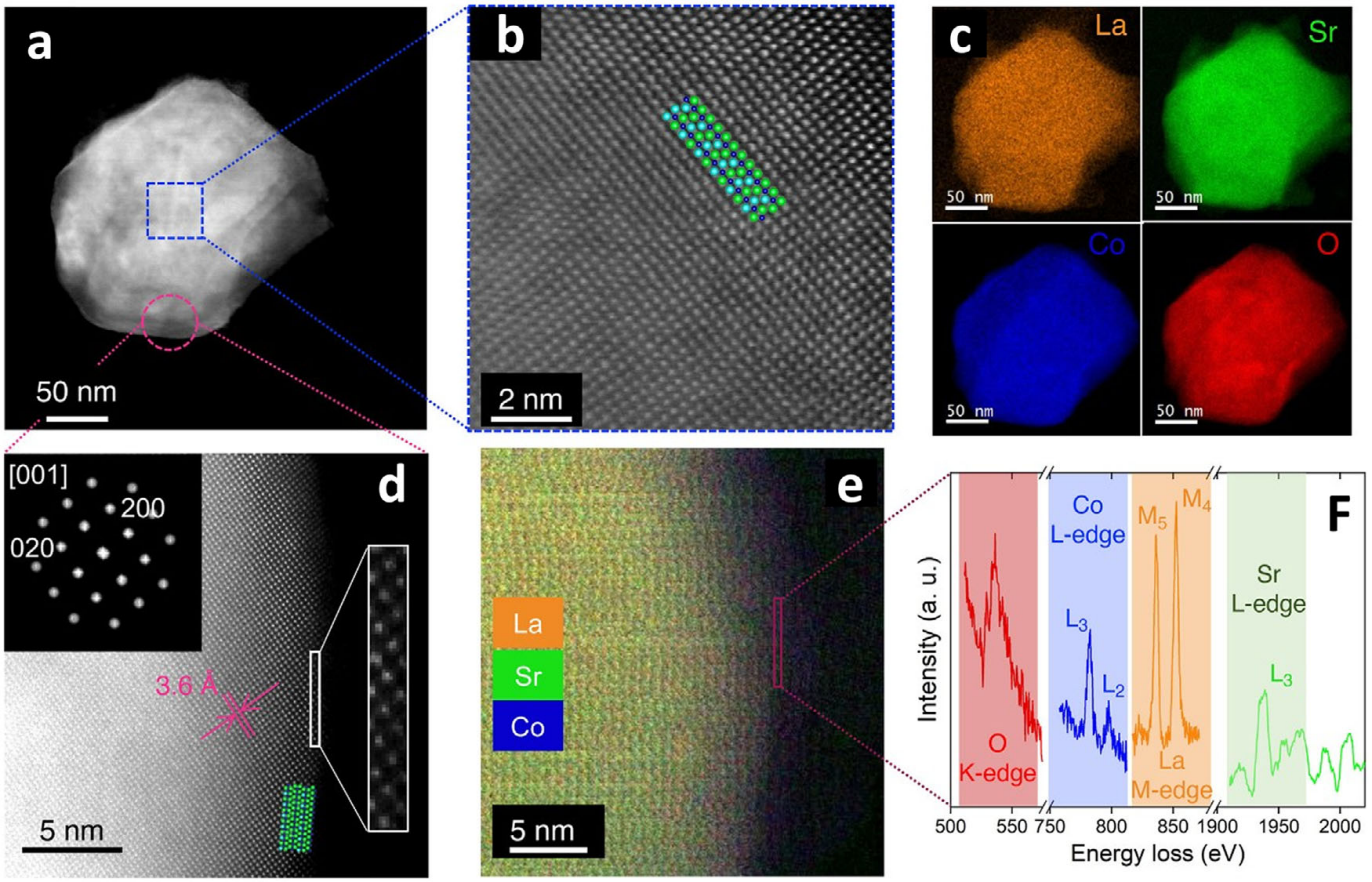
Atomic‐scale structural and chemical characterization of pristine LSCoO‐75 (La_0_._8_Sr_0_._2_CoO_3_−δ) prior to electrochemical cycling. (a) HAADF‐STEM image showing particle morphology. (b,d) Atomic‐resolution HAADF‐STEM images of bulk and near‐surface regions, revealing continuous lattice fringes (∼3.6 Å) consistent with the Ruddlesden–Popper (I4/mmm) structure along the [001] direction. (c,e) EELS elemental mapping demonstrating uniform distribution of La, Sr, Co, and O throughout the particle and near‐surface region. (f) Averaged EELS spectra confirming the expected chemical states and absence of impurity phases [84]. Adapted with permission from Ref. [84]. Copyright 2021, ACS.

Another important consideration is dynamic surface evolution under operating conditions. Samira et al. (2021) demonstrated that surface reconstruction during electrochemical reactions can lead to the formation of active oxyhydroxide layers through cation dissolution processes [84]. The low‐magnification high‐angle annular dark‐field scanning transmission electron spectroscopy (HAADF‐STEM) image of pristine LSCoO‐75 (La_0_._8_Sr_0_._2_CoO_3_−δ) is depicted in Figure 8a, which means the catalyst consists of uniform polyhedral particles with no visible defects or surface contamination at the micrometer scale. Figure 8b shows an atomic‐resolution HAADF‐STEM image of the same material, which means continuous lattice fringes extend all the way to the particle surface with a measured spacing of ∼3.6 Å along the [001] direction, confirming high crystallinity and the absence of surface amorphization or reconstruction in the pristine state. Electron energy loss spectroscopy (EELS) elemental maps of La, Sr, and Co over a 50 nm region are shown in Figure 8c, representing the spatial distribution of cations within both the bulk and near‐surface areas. This mapping was performed to assess cation segregation, a common issue in perovskite oxides where Sr or La can migrate to the surface. A homogeneous distribution is observed, confirming that no such segregation occurs in the pristine state and thereby establishing a chemically uniform baseline. Figure 8d provides a higher‐magnification atomic‐resolution HAADF‐STEM image (scale bar: 2 nm), which represents the atomic‐scale structure of the surface region. This image was collected to check for any surface amorphization, reconstruction, or disorder. The continuous lattice fringes extending sharply to the outermost atoms confirm a well‐ordered Ruddlesden–Popper (I4/mmm) structure with unreconstructed terminations, proving the surface is structurally pristine.

High‐resolution EELS maps (scale bar: 5 nm) of La, Sr, and Co are presented in Figure 8e, representing the elemental distribution in the near‐surface region (within a few nanometers of the edge). These maps were acquired to assess subtle cation enrichment or depletion that may not be detectable at lower resolution. A uniform signal is observed even at this scale, reinforcing the absence of any compositional gradient and confirming chemical homogeneity down to the outermost atomic layers. Figure 8f shows the averaged EELS spectra from multiple regions, which represent the chemical bonding states and electronic structures of all constituent elements. This spectrum was collected to check for impurity phases, oxidation state anomalies, or unexpected chemical environments. The characteristic energy losses Co L‐edge (∼780 eV), La M‐edge (∼850 eV), Sr L‐edge (∼1,950 eV), and O K‐edge (∼530 eV) appear at their expected positions, confirming that all elements are present in their correct chemical states with no detectable impurities. Together, these four panels establish that the as‐synthesized LSCoO‐75 is structurally coherent (no reconstruction, Figure 8d) and chemically uniform (no segregation, Figure 8c,e,f). Therefore, any catalytic activity observed under operating conditions must arise from in situ surface reconstruction, such as oxyhydroxide formation via cation dissolution rather than from any pre‐existing surface feature of the pristine material.

Importantly, the impact of these design strategies is reflected in electrochemical performance trends summarized in Table 3, which compiles representative REE‐based materials across cathodes, separators, binders, and adsorbents. The data reveal several key patterns. First, systems incorporating conductive frameworks (e.g., carbon‐supported REEs or hybrid composites) consistently exhibit improved rate capability and cycling stability. Second, materials combining adsorption and catalytic functions, such as heterostructures and defect‐engineered oxides tend to outperform systems relying on a single mechanism. Third, significant variability in sulfur loading, testing conditions, and cell configurations complicates direct comparison, emphasizing the need for standardized evaluation metrics.

**TABLE 3:** Summary of electrochemical performance metrics for REE‐based Li–S battery materials.

Material	REE used	Application	Performance metric	Value	Refs.
GB‐Y binder	Y	Cathode binder	Initial capacity	1201.5 mAh g^−1^	[56]
			Sulfur loading	6.56 mg cm^−2^	
La@Sn_3_O_4_/CeO_2_/S	La, Ce	Cathode	Initial capacity (0.1 C)	1560 mAh g^−1^	[85]
			Decay rate (500 cycles, 1 C)	0.09% per cycle	
La_2_O_2_S‐C/PP	La	Separator	Capacity after 300 cycles (1 C)	664 mAh g^−1^	[57]
Sm‐N_3_C_3_	Sm	Separator	Half‐cell capacity	>600 mAh g^−1^	[58]
			Half‐cell retention (2000 cycles)	84.3%	
Nd_2_O_3_/RGO/PP	Nd	Separator	Decay rate (1000 cycles, 2 C)	0.0525% per cycle	[59]
			Rate performance (3 C)	614 mAh g^−1^	
TM‐RE HEO	Nd, Gd, Sm, Pr	Functional interlayer	Initial capacity (0.1 C)	1146 mAh g^−1^	[86]
			Decay rate (300 cycles, 0.5 C)	0.08% per cycle	
Ce_2_O_2_S/C	Ce	Sulfur carrier	Enhanced adsorption	S─S and Li─S bond formation	[87]
CMC	La, Dy, Lu	Adsorbent	Adsorption enhancement	2–4× pristine carbon	[88]
(P‐AM‐AA)/AC	Ce, Nd, Gd	Adsorbent	Sorption efficacy	>98%	[89]
Carbon‐ferrite	La, Sm, Nd, Pr	Preconcentration	Preconcentration factor	141–246	[90]
OP‐CN	La, Ce, Gd, Yb	Adsorbent	Adsorption capacity	76.2–111.7 mg g^−1^	[91]
LaMOF‐GO_3_	La	Composite	Surface area increase	14.8 → 26.6 m^2^ g^−1^	[92]
Y(BTC)(H_2_O)·(DMF)	Y	Adsorbent	Sulfur adsorption capacity	30.7 mg S/g	[93]
La@BTC	La	Adsorbent	Defluoridation capacity	4985 mg F^−^ kg^−1^	[94]
Ce@BTC	Ce	Adsorbent	Defluoridation capacity	4930 mg F^−^ kg^−1^	

From these observations, several overarching design principles can be identified. Effective REE‐based materials require: (i) conductive integration to ensure electron transport, (ii) balanced adsorption–conversion behavior to avoid passivation, (iii) defect and electronic structure engineering to optimize catalytic activity, and (iv) structural adaptability to accommodate dynamic changes during cycling. In this context, REEs should not be viewed merely as strong adsorbents, but as versatile electronic modifiers capable of regulating interfacial reactions at multiple scales. These insights collectively indicate that the performance of REE‐based materials arises from the interplay of electronic structure, surface chemistry, and composite architecture rather than any single intrinsic property. As the field moves toward practical Li–S systems, the emphasis should shift from empirical material development to descriptor‐guided design, where REE selection and structural engineering are systematically linked to catalytic function and device‐level performance.

The diversity of REE‐based composite architectures (carbon, MOF, oxide, heterostructure) raises a fundamental question: which structural features are truly essential, and which are incidental? Current literature provides no clear answer because direct comparisons are confounded by variations in synthesis, testing conditions, and REE identity. A critical gap is the absence of systematic structure–property maps that isolate the contribution of the REE itself. Furthermore, most studies report performance under idealized conditions (low sulfur loading, excess electrolyte), making it impossible to assess whether complex composite architectures are necessary or whether simpler designs would suffice. Future work should employ design‐of‐experiments approaches and report failure rates alongside best‐case performance.

## 4 Functional Roles of Rare‐Earths Across Cell Components

The effective utilization of REEs in Li–S batteries requires their strategic deployment across multiple cell components. Unlike conventional catalyst systems, where functionality is typically localized at a single interface, REE‐based materials enable multifunctional and spatially distributed design, in which cathode, separator, electrolyte, and anode each contribute distinct yet interconnected roles. This system‐level perspective is particularly important for Li–S batteries, where performance is governed by coupled phenomena including polysulfide transport, interfacial reactions, and lithium deposition. REEs offer a unique advantage in this context due to their ability to simultaneously modulate adsorption, catalytic conversion, and interfacial electronic structure. Rather than acting solely as active sites, REE species can function as interfacial regulators, influencing reaction pathways, ion transport, and chemical stability across the entire cell architecture. The following sections examine these roles in a component‐specific manner, beginning with the cathode, followed by separator and electrolyte interfaces, and finally the lithium metal anode, ultimately highlighting opportunities for integrated REE‐based cell design.

### 4.1 Cathode Hosts and Electrocatalysts

The cathode represents the primary site of sulfur redox reactions and remains the most extensively studied domain for REE integration. Effective cathode design requires balancing three interdependent functions: (i) adsorption of soluble polysulfides to suppress shuttling, (ii) catalytic acceleration of sulfur conversion reactions, and (iii) efficient electron transport. These requirements often impose conflicting constraints. Strong adsorption can immobilize intermediates and hinder conversion, while highly conductive carbon materials lack sufficient chemical affinity to retain polysulfides. REE‐based materials provide a pathway to reconcile these trade‐offs by combining strong polarity with tunable catalytic activity.

Single‐atom REE catalysts represent one of the most effective strategies for maximizing active site utilization and ensuring uniform catalytic behavior. For example, Wei et al. engineered a three‐dimensional nitrogen‐doped carbon framework with atomically dispersed cerium sites (3DCeSA‐N‐WS), where a “micro‐region welding” strategy created continuous conductive pathways between carbon nanosheets and nanotubes [39]. This architecture enables efficient electron transport while exposing isolated Ce active sites for polysulfide adsorption and catalytic conversion, demonstrating how atomic dispersion and conductive integration can synergistically enhance performance.

In addition to atomically dispersed systems, surface reconstruction and overlayer formation induced by REE incorporation can significantly influence catalytic activity. Studies on rare‐earth–modified Pt alloys (e.g., Pt–Y and Pt–Gd) have shown that REE alloying can drive the formation of strained metallic overlayers with enhanced catalytic properties [95]. Although these systems are not specific to Li–S batteries, they provide important insight into how REEs can modify surface electronic structure and induce dynamic reconstruction, suggesting analogous mechanisms may operate in sulfur redox environments.

Defect engineering combined with REE doping offers another effective route for enhancing catalytic activity. Tian et al. (2025) reported Ce‐doped NiS_2_ nanosheet catalysts supported on carbon cloth, where the introduction of sulfur vacancies and Ce dopants synergistically modulated local charge distribution and promoted surface reconstruction [96]. In situ Raman spectroscopy and DFT analysis revealed that these modifications facilitate the formation of catalytically active species and accelerate reaction kinetics. While this system was studied in the context of OER and UOR, the underlying principle of defect‐mediated electronic modulation coupled with REE doping is directly relevant to improving polysulfide conversion in Li–S batteries.

These examples highlight that REE functionality in cathode systems extends beyond simple adsorption enhancement. Instead, REEs influence catalytic behavior through a combination of atomic dispersion, electronic structure modulation, defect engineering, and dynamic surface reconstruction. However, a persistent challenge is the difficulty in distinguishing intrinsic REE effects from contributions arising from conductive supports, heterostructure interfaces, or defect states. Addressing this issue will require carefully designed model systems and operando characterization to isolate the true catalytic role of REEs in sulfur redox chemistry.

#### 4.1.1 Li_2_S Deposition: The Rate‐Determining Step in Discharge

Beyond polysulfide adsorption and conversion, the nucleation and growth of solid Li_2_S from soluble LiPSs) represents the final and most kinetically challenging step in the discharge process. This solid‐state transformation accounts for approximately 75% of the theoretical capacity of the sulfur cathode, yet its sluggish kinetics stemming from high interfacial impedances between the electrolyte and the substrate often leads to incomplete sulfur utilization, premature cell death, and poor rate capability. Consequently, the ability of a catalyst to lower the Li_2_S nucleation overpotential and promote uniform, high‐density Li_2_S deposition is a definitive measure of true electrocatalytic activity, distinguishing it from mere polysulfide adsorption.

Recent studies have established quantitative frameworks for evaluating this critical process. The electrodeposition of Li_2_S follows overpotential‐driven progressive and instantaneous nucleation mechanisms, where a lower overpotential (typically below 50 mV for catalytic surfaces) correlates with higher nucleus density and more uniform film growth. The Li_2_S decomposition energy barrier is directly associated with the binding between isolated Li ions and sulfur in sulfides. This is the main reason catalytic materials induce lower overpotential compared to conventional carbon hosts. Therefore, measuring the Li_2_S nucleation overpotential and comparing it to non‐catalytic controls provides a quantitative metric to distinguish adsorption‐dominated materials from true catalysts.

The significance of Li_2_S deposition has been extensively discussed in the literature. Yin et al. reviewed the industrialization of Li_2_S nano‐powder material, highlighting that the high activation potential and poor electronic/ionic conductivity of Li_2_S remain major obstacles to its practical application as a cathode material in Li‐S batteries [97]. They emphasized that reducing the activation potential of Li_2_S through appropriate electrolyte additives not only enhances rate capability but also improves cycling performance. Complementing this perspective, Li et al. systematically demonstrated that introducing suitable electrolyte additives can effectively lower the activation potential of Li_2_S, thereby promoting rate capability and cycling stability [98]. This perspective underscores that Li_2_S activation is a bottleneck that must be addressed through rational materials design, including rare‐earth‐based catalysts.

For rare‐earth‐based catalysts, emerging evidence indicates that their unique 4f electronic configurations offer distinct advantages in promoting Li_2_S nucleation. Vacancy‐engineered CeO_2_ has been shown to lower the barriers for Li_2_S nucleation and decomposition through vacancy‐induced orbital hybridization between Ce‐4f/S‐3p and Li‐2s/O‐2p states [40].

This electronic modulation not only enhances LiPS adsorption but also accelerates bidirectional sulfur conversion, enabling stable cycling (743.2 mAh g^−1^ after 1000 cycles at 0.5 C) and high‐rate capability (up to 5 C). Similarly, the construction of rare‐earth‐based heterostructures, such as La@Sn_3_O_4_/CeO_2_, provides defect‐rich and highly active interfaces that promote the catalytic conversion of LiPSs to Li_2_S, as confirmed by electrochemical tests and in situ Raman characterization [85]. This work demonstrates that the unique 4f5d electron orbitals and variable valence states of rare‐earth elements effectively enhance catalytic efficiency toward Li_2_S formation.

The orbital coupling strategy has further advanced the understanding of Li_2_S deposition kinetics. The incorporation of Eu 4f orbitals into NiSe induces gradient d‐p‐f orbital coupling, reconstructing both Ni 3d and Se 4p states [46]. This electronic reconstruction strengthens hybridization between the active sites and LiPSs, thereby optimizing the band center alignment and lowering the energy barrier for Li_2_S conversion. The resulting Eu‐incorporated NiSe catalyst delivers exceptional electrochemical performance, with a high specific capacity of 896.2 mAh g^−1^ at 4 C and a retained areal capacity of 5.66 mAh cm^−2^ under a high sulfur loading of 5.94 mg cm^−2^ after 100 cycles.

Beyond rare‐earth‐based systems, the dynamic evolution of catalyst surfaces during Li_2_S deposition has been directly visualized in transition metal catalysts. Cheng et al. demonstrated that cobalt nanoparticles undergo surface conversion to cobalt sulfide during electrochemical reactions, forming a Co@Co_x_S_γ_ heterostructure that significantly enhances Li_2_S conversion kinetics [99]. This catalytic surface evolution enabled a reversible capacity of 1133 mAh g^−1^ over 1000 cycles at 1C. This finding underscores the importance of understanding catalytic surface restructuring a phenomenon that may also occur in REE‐based catalysts under operating conditions, where the 4f electronic configuration could drive analogous dynamic transformations. Future operando studies should therefore monitor not only the polysulfide intermediates but also the surface chemistry of REE catalysts during Li_2_S nucleation.

These studies establish that Li_2_S nucleation overpotential and decomposition barrier are critical metrics for evaluating rare‐earth‐based electrocatalysts. A genuine REE catalyst reduces the nucleation overpotential (typically below 50 mV), promotes high‐density uniform Li_2_S deposition, and lowers the decomposition energy barrier, thereby enabling complete sulfur utilization under practical conditions. Future research on REE‐based Li–S batteries should therefore include systematic Li_2_S nucleation and decomposition experiments alongside conventional electrochemical characterization to provide a complete picture of catalytic activity.

### 4.2 Interlayer/Separator Modifiers

Separator and interlayer engineering play a critical role in regulating polysulfide transport and interfacial reactions in Li–S batteries. Unlike cathode design, which focuses on reaction kinetics and sulfur utilization, separator modification primarily aims to block polysulfide migration while maintaining ion transport, thereby suppressing shuttle effects without introducing additional resistance. Rare earth element (REE)‐based layered materials are particularly well‐suited for this purpose due to their tunable interlayer chemistry, strong polarity, and ability to host dynamic ion–molecule interactions. Layered rare‐earth compounds with exchangeable interlayer species provide a versatile platform for separator modification. As reviewed by Geng et al., anion‐exchangeable layered rare‐earth phosphors exhibit flexible interlayer environments, where lanthanide contraction influences lattice spacing, coordination geometry, and structural stability [100]. Water molecules coordinated to REE ions extend into the interlayer galleries, stabilizing the layered framework and enabling reversible hydration–dehydration transitions. In the context of Li–S batteries, such dynamic interlayer environments can facilitate Li^+^ transport while simultaneously restricting the diffusion of larger polysulfide species.

The role of interlayer chemistry is further highlighted by studies on layered rare‐earth hydroxides. Kim et al. demonstrated that interlayer anions can significantly influence functional properties by acting as filters, spacers, or sensitizers depending on their interaction with REE centers [101]. Although originally explored for photophysical behavior, this concept is directly transferable to Li–S systems: interlayer species can regulate polysulfide transport by modifying electrostatic interactions, steric confinement, and local chemical environments within the separator. Ion transport and confinement behavior within layered structures are also strongly dependent on REE identity. Molecular dynamics simulations by Wang et al. showed that both light (La^3+^) and heavy (Lu^3+^) rare earth ions adopt stable coordination environments within layered hosts, forming outer‐sphere complexes with reduced mobility compared to aqueous systems [102]. This reduced mobility suggests that REE‐modified interlayers can effectively immobilize charged species, providing a mechanism for suppressing polysulfide diffusion while maintaining structural stability.

Structural tunability through interlayer spacing further expands the design space for separator materials. Feng et al. demonstrated that layered rare‐earth hydroxides (LRHs) can achieve a wide range of basal spacings from 8.3 to 46 Å depending on the interlayer anion [103].

**FIGURE 9:**
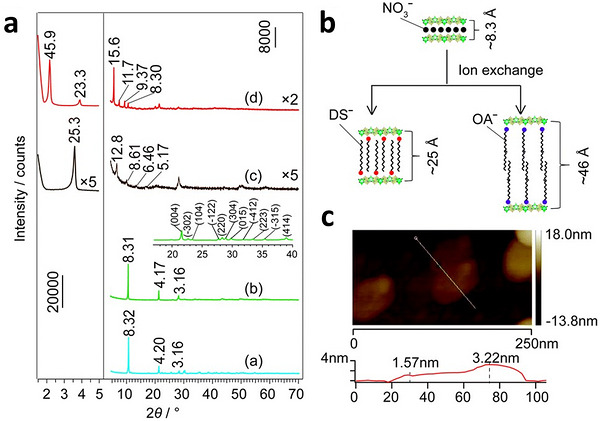
Tunable interlayer structure in layered rare‐earth hydroxides (LRHs) and its impact on ion transport properties. (a) XRD patterns of LRHs intercalated with different anions: Cl^−^ (basal spacing 8.3 Å), NO_3_^−^ (∼8.9 Å), SO_4_^2−^ (∼11 Å), and dodecyl sulfate (DS^−^, ∼46 Å). The progressive shift of the (00l) peaks to lower 2θ angles indicates increasing interlayer distance. (b) Schematic of the LRH structure: positively charged rare‐earth hydroxide host layers (e.g., [RE_2_(OH)_5_·Nh_2_O]^+^) with exchangeable anions and water molecules in the interlayer gallery. As the anion size increases (from Cl^−^ to DS^−^), the gallery expands, creating larger nanochannels. (c) Illustration of size‐selective transport: small Li^+^ ions can diffuse through narrow interlayer spaces (∼8 Å), while larger polysulfide anions (Li_2_S_4_, ∼4–6 Å; Li_2_S_6_, ∼8–12 Å) are physically blocked when the spacing is tuned below their effective size. This tunability enables rational design of REE‐based separators that balance Li^+^ conductivity with polysulfide confinement. Adapted with permission [103]. Adapted with permission from Ref. [103]. Copyright 2018, RSC.

The X‐ray diffraction patterns in Figure 9a demonstrate that the basal spacing of layered rare‐earth hydroxides (LRHs) can be systematically tuned by interlayer anion exchange. As smaller anions (e.g., Cl^−^) are replaced with larger ones (e.g., NO_3_^−^, SO_4_^2−^, and dodecyl sulfate), the diffraction peaks shift to lower angles, indicating a progressive expansion of the interlayer distance. This structural tuning is intentionally performed to modulate the size of the interlayer galleries, which act as ion transport pathways. To visualize how this expansion translates into actual layer geometry, Figure 9b provides a corresponding structural model that illustrates the arrangement where positively charged rare‐earth hydroxide layers are separated by exchangeable anions and intercalated water molecules. The interlayer spacing quantified in Figure 9a directly translates here into the gallery height between adjacent layers. As the anion size increases, the structure evolves from a tightly confined interlayer region to a more open, nanochannel‐like architecture, providing a clear structural basis for controlling mass transport. Figure 9c shows how this structural evolution governs transport behavior. The confined galleries at smaller spacings effectively suppress the diffusion of large lithium polysulfide species, enhancing their confinement. In contrast, expanded interlayers facilitate faster ionic transport by reducing steric hindrance, but at the cost of weaker polysulfide trapping. This approach is intentionally used to balance two competing requirements in Li–S systems, effective polysulfide confinement and efficient ionic transport, thereby identifying interlayer spacing as a critical structural descriptor for optimizing separator performance.

Beyond oxide‐based systems, layered chalcogenides also exhibit promising ion‐exchange and adsorption properties. Qi et al. reported a layered SnS‐based material with exceptional ion‐exchange capacity, fast kinetics, and high selectivity for rare earth cations [104]. While primarily developed for REE recovery, these properties suggest that similar materials could be adapted for Li–S separators to selectively trap polysulfides and regulate ionic transport. However, their direct application in electrochemical environments remains to be explored. Finally, systematic studies on rare‐earth adsorption behavior provide insight into how ionic size influences interlayer interactions. Takei et al. demonstrated that the position and coordination environment of REE cations within layered materials depend strongly on ionic radius, with larger ions occupying central interlayer positions and smaller ions residing closer to host layers [105]. These findings indicate that lanthanide selection can be used as a design parameter to control interlayer structure and interaction strength with charged species.

### 4.3 Electrolyte Additives and Anode Protection

Rare‐earth‐based electrolyte additives have emerged as effective strategies for stabilizing metal anodes and regulating interfacial reactions in Li–S and related battery systems. Unlike cathode or separator modifications, which primarily target polysulfide confinement and conversion, electrolyte additives operate at the electrode–electrolyte interface, where they influence solvation structure, interfacial chemistry, and metal deposition behavior. One of the key functions of REE additives is the formation of protective interfacial layers that suppress side reactions. For example, Wang et al. (2015) demonstrated that organic–rare earth complexes formed from l‐cysteine and cerium nitrate can generate protective films on aluminum alloy anodes, reducing corrosion, suppressing hydrogen evolution, and improving anodic utilization [106]. Although studied in aluminum–air systems, this mechanism is directly relevant to Li–S batteries, where interfacial stability and parasitic reactions similarly limit performance.

**FIGURE 10:**
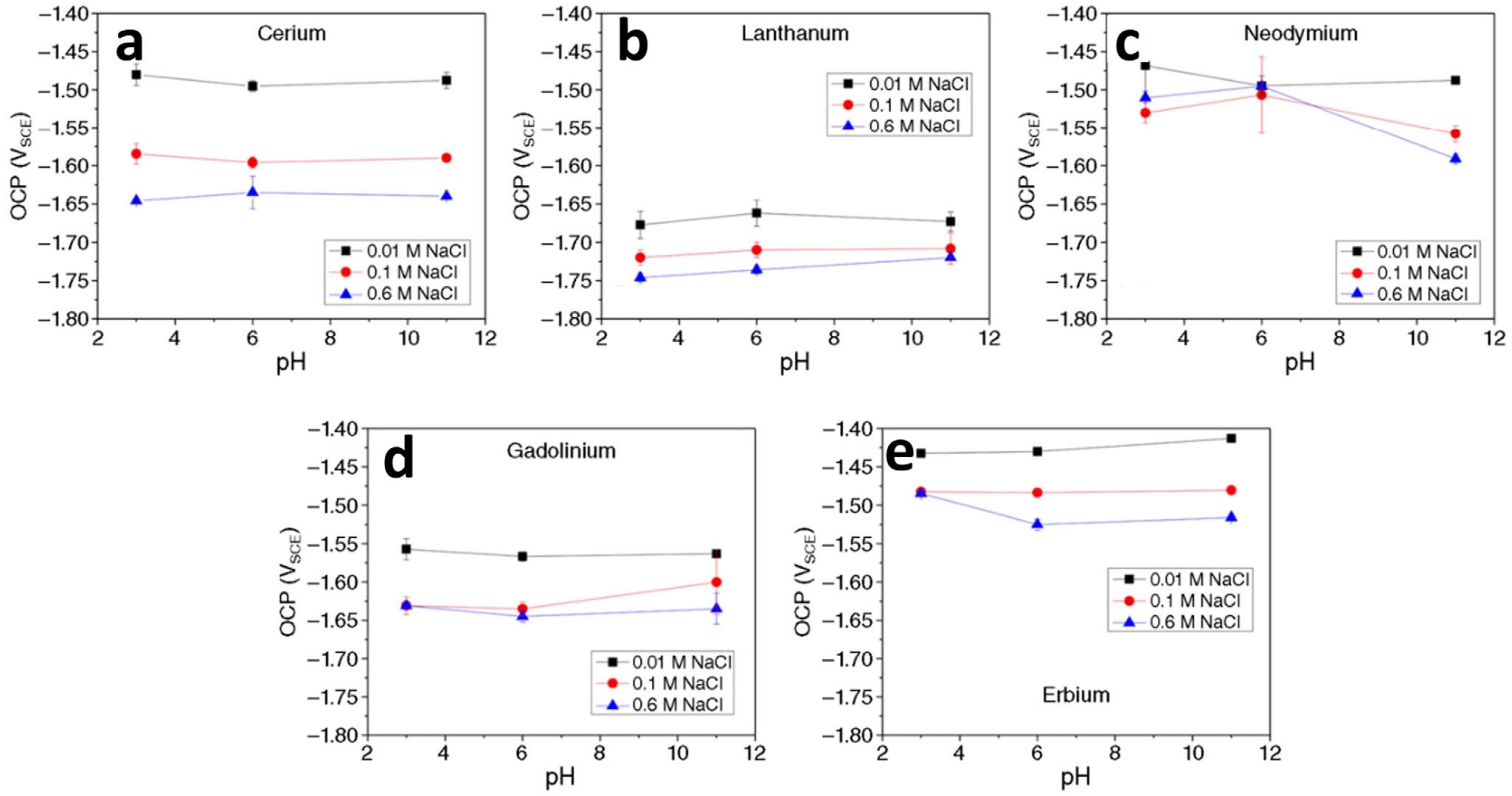
OCP measurements of five rare earth elements (Ce, La, Nd, Gd, Er) as a function of pH (2–12) and NaCl concentration (0.01, 0.1, and 0.6 M). (a–e) OCP values for Ce, La, Nd, Gd, and Er, respectively. Increasing chloride concentration shifts the OCP to more negative values for all REEs, indicating enhanced anodic dissolution in Cl^−^‐containing environments. In contrast, OCP values show relatively weak dependence on pH across the investigated range. A systematic trend across the lanthanide series is observed, with Er exhibiting the most noble (least negative) behavior and La the most active (most negative), following the order Er > Nd > Ce > Gd > La. These trends reflect intrinsic differences in surface oxide stability and dissolution kinetics, providing a rational basis for selecting REE additives based on chloride exposure [109]. Adapted with permission from Ref. [109]. Copyright 2014, AMPP.

More advanced strategies involve dual‐functional electrolyte additives that simultaneously regulate chemical and electrostatic environments. Li et al. (2024) developed a water‐insoluble yttrium‐based additive (YTFPAA), which stabilizes metal anodes through multiple mechanisms, including proton capture, dynamic surface adsorption, and redistribution of ion flux [107]. These combined effects lead to the formation of a water‐deficient interfacial region that suppresses side reactions and enhances deposition reversibility, illustrating how REEs can actively modulate solvation and interfacial structure. Electrostatic shielding has also emerged as a key mechanism for dendrite suppression. Wang et al. introduced Nd^3+^ ions as electrolyte additives, showing that preferential adsorption on specific crystal planes can create a positively charged shielding layer that regulates ion deposition and promotes uniform growth [108]. This strategy is particularly relevant for Li metal anodes in Li–S batteries, where dendrite formation and uneven deposition remain critical challenges. Figure 10a–e presents open‐circuit potential (OCP) measurements for five representative rare earth elements Ce, La, Nd, Gd, and Er, as a function of pH (2–12) and NaCl concentration (0.01 M, 0.1 M, and 0.6 M). These measurements were collected to determine how electrolyte composition influences the intrinsic corrosion behavior of REEs when used as additives. Specifically, Figure 10a presents OCP data for cerium (Ce), which shows a shift from −1.540 to −1.510 V across pH 2–12 at 0.01 M NaCl, indicating a modest noble shift with increasing pH. At 0.6 M NaCl, the OCP becomes significantly more negative (e.g., −1.650 V at pH 2), indicating that high chloride concentration strongly promotes anodic dissolution. Figure 10b presents OCP data for lanthanum (La), which exhibits more negative potentials overall (e.g., −1.640 V at pH 2, 0.01 M NaCl) compared to cerium, indicating a more active (less noble) electrochemical behavior. Increasing NaCl concentration to 0.6 M shifts the OCP negatively by approximately 0.11 V, confirming high susceptibility to chloride‐induced corrosion. Figure 10c presents OCP data for neodymium (Nd), which ranges from −1.580 to −1.570 V at 0.01 M NaCl across pH 2–12, indicating relatively stable potential across pH. At 0.6 M NaCl, the OCP reaches −1.605 V at pH 10, indicating enhanced anodic dissolution, with neodymium showing intermediate behavior between lanthanum (more active) and erbium (more noble). Figure 10d presents OCP data for gadolinium (Gd), which ranges from −1.540 to −1.560 V at 0.01 M NaCl across pH 2–12, indicating little pH dependence. Increasing chloride to 0.6 M shifts the OCP negatively to approximately −1.570 V, with gadolinium exhibiting slightly more negative potentials than neodymium, representing the second most active behavior among the five REEs. Figure 10e presents OCP data for erbium (Er), which ranges from −1.500 V (pH 2) to −1.460 V (pH 12) at 0.01 M NaCl, representing the most noble (least negative) potentials among all five REEs. Even at 0.6 M NaCl, the OCP remains relatively positive (e.g., −1.520 V at pH 2), indicating that erbium is intrinsically more resistant to anodic dissolution in chloride‐containing environments [109].

Together, Figure 10a–e establishes a systematic trend across the lanthanide series: Er > Nd > Ce > Gd > La from most noble to most active. This trend represents intrinsic differences in surface oxide stability and dissolution kinetics, providing a rational basis for selecting REE additives based on expected chloride exposure.

Moreover, REEs can also function as dynamic interfacial regulators that simultaneously influence multiple cell components. For example, europium‐based additives have been shown to form electrostatic shielding layers that control ion deposition while suppressing parasitic reactions, leading to improved cycling stability in full‐cell configurations [110]. Similarly, rare‐earth doping of anode materials can enhance intrinsic properties such as ion diffusion and structural stability, as demonstrated in La‐doped NaTi_2_(PO_4_)_3_ systems, where improved diffusion kinetics and long‐term cycling stability were achieved [111]. Importantly, the diverse roles of REEs across electrolyte and anode systems are reflected in the performance metrics summarized in Table 4. The table highlights a wide range of strategies, including electrolyte additives, anode coatings, alloyed anodes, and gel polymer electrolytes, demonstrating consistent improvements in cycle life, Coulombic efficiency, and ion transport behavior. However, significant variability in testing conditions, battery chemistries, and evaluation protocols complicates direct comparison, emphasizing the need for standardized benchmarks and Li–S‐specific validation.

**TABLE 4:** Summary of electrochemical performance metrics for REE‐based modifications across cell components.

Material/Strategy	REE used	Application	Performance metric	Value	Refs.
La_0_._8_Gd_0_._2_MnO_3_	Gd	Cathode catalyst	Mass activity enhancement	2.4× vs. LaMnO_3_	[112]
			Turnover frequency enhancement	4.3× vs. LaMnO_3_	
SmFeO_x_@CN	Sm	ORR catalyst	Activity comparable to	Pt/C	[113]
Y^3+^‐controlled GO membrane	Y	Ion separation	Separation factor (Sc/Y)	4.02	[114]
Sm(OAc)_3_/Yb(OAc)_3_	Sm, Yb	Electrolyte additive	Capacity density improvement	9.6–16.3%	[115]
			Optimal concentration	200 mg/L	
Y(NO_3_)_3_	Y	Electrolyte additive	Li//Li symmetric cell life	>1000 h at 1 mA cm^−2^	[116]
Nd(OTf)_3_	Nd	Electrolyte additive (Li–S)	Areal capacity	5.5–7.0 mAh cm^−2^	[117]
			Coulombic efficiency	94%–95%	
YTFPAA	Y	Electrolyte additive	Zn//Zn cycle life	2100 h at 0.5 mA cm^−2^	[107]
			V_2_O_5_//Zn full cell cycles	18,000 cycles at 5 A g^−1^	
CeN_3_O_9_·6H_2_O	Ce	Electrolyte additive	Corrosion inhibition rate	70.4%	[118]
			Charge transfer resistance increase	69.5 Ω	
			Discharge time extension	40 min	
Y^3+^‐V_2_O_5_·nH_2_O	Y	Cathode (ZIB)	Capacity at 500 mA g^−1^	337 mAh g^−1^	[119]
			Capacity at 10 A g^−1^	170 mAh g^−1^	
			Capacity retention (3000 cycles)	90%	
			Interlayer spacing	13.6 Å	
CPAM‐LC GPE	La, Ce	Gel polymer electrolyte	Ionic conductivity	327 mS cm^−1^	[120]
			Power density	203.4 mW cm^−1^	
			Charge–discharge cycles	>1300	
Yb additive	Yb	Anode additive	Zn//Zn cycle life	2400 h at 1 mA cm^−2^	[121]
			Cycle life at 80% Zn utilization	125 h	
			Full cell cycles	1000 cycles at 5 A g^−1^	
			Pouch cell stability	65 cycles	
Zn–Ce/Yb alloy	Ce, Yb	Alloyed anode	Dendrite inhibition efficiency	35%	[122]
RCl_3_ additives	Various	Electrolyte additive	Zn//Zn cycle life	>8000 cycles at 5 mA cm^−2^	[123]
			Zn utilization	68.3% for 130 h	
			Pouch cell capacity retention	Nearly 100% after 300 cycles	
PY150@Zn coating	Y	Anode coating	Zn//Zn cycle life	>4100 h at 1 mA cm^−2^	[124]
Y^3+^ in ZnSO_4_	Y	Electrolyte additive	Zn//Zn cycle life	2080 h at 5 mA cm^−2^	[125]
			Zn//NH_4_V_4_O_10_ retention	89.6% after 2000 cycles	

From a design perspective, several key principles emerge. Effective REE‐based electrolyte strategies rely on: (i) interfacial stabilization through protective film formation, (ii) electrostatic regulation to control ion flux and deposition behavior, (iii) solvation structure modulation to suppress parasitic reactions, and (iv) multifunctional integration across cathode, electrolyte, and anode interfaces. In this context, REEs should be viewed not merely as additives, but as active regulators of interfacial chemistry capable of orchestrating coupled processes across the entire cell. Rather than functioning in isolation, the most promising approaches involve coordinated REE deployment across multiple components, where synergistic effects lead to performance improvements that cannot be achieved through single‐component modification alone. This integrated strategy represents a critical direction for advancing Li–S battery technology toward practical implementation.

While REE‐based electrolyte additives and anode protection strategies show promise, the field suffers from a lack of mechanistic specificity. Many studies report improved cycle life but do not identify whether the REE additive participates in SEI formation, acts as an electrostatic shield, or simply scavenges trace impurities. A direct contradiction exists: some reports claim that La^3+^ additives promote uniform Li deposition, while others find that La^3+^ accelerates corrosion. These contradictory findings likely arise from differences in electrolyte composition, current density, and cell configuration, but no systematic study has resolved them. Additionally, nearly all REE additive work has been done in Li||Li symmetric cells or Li||LiFePO_4_ systems, not in practical Li–S pouch cells with high sulfur loading. Validation in realistic Li–S environments is urgently needed.

## 5 Rare‐Earth Functional Deployment Across Cell Components

The preceding sections demonstrate that REEs possess a unique combination of properties, including strong Lewis acidity, variable oxidation states, and distinctive 4f electronic structures that enable effective interaction with sulfur species. However, the true potential of REEs in Li–S batteries lies not in isolated material design, but in their coordinated deployment across the entire cell architecture. In this context, REEs should be viewed as multifunctional regulators rather than single‐role catalysts. Their functionality can be spatially distributed across the cathode, separator, electrolyte, and anode, where each component leverages a specific aspect of REE chemistry, ranging from catalytic conversion and adsorption at the cathode to ion regulation and interfacial stabilization at the separator and anode. This system‐level strategy is particularly important for Li–S batteries, where performance is governed by strongly coupled processes such as polysulfide transport, reaction kinetics, and lithium deposition. Recent advances in catalytic materials further illustrate the importance of integrated design. For example, Zhen et al. (2025) developed a dual‐site catalytic architecture combining cobalt single atoms and nanoclusters on nitrogen‐doped MXene, where electronic modulation and interfacial charge redistribution significantly enhanced polysulfide conversion and lithium deposition behavior [126]. Although not REE‐based, this work highlights a broader principle: multiscale and multifunctional catalyst design is essential for achieving high performance under practical conditions. Translating such concepts to REE systems provides a pathway toward fully optimized Li–S batteries.

### 5.1 REE in Sulfur Cathodes

The sulfur cathode remains the primary platform for REE deployment, where their role is to balance polysulfide adsorption, catalytic conversion, and electronic conductivity. Rather than relying on a single mechanism, recent studies demonstrate that synergistic integration of multiple functionalities is critical for achieving high performance. Perovskite‐based systems represent one effective strategy for combining catalytic activity with structural stability. For instance, Hong et al. (2024) developed LaNi_0_._6_Co_0_._4_O_3_ hollow porous nanofibers as bidirectional sulfur hosts, where Co substitution induced oxygen vacancies and enhanced electrical conductivity while the B‐site transition metals facilitated polysulfide conversion [127]. This system highlights how defect engineering and transition‐metal synergy can amplify the catalytic role of REEs within complex oxide frameworks.

Electronic structure tuning provides another powerful design approach. Duan et al. demonstrated that bandgap engineering in quantum dot‐based systems can directly influence adsorption–catalytic behavior, with optimized electronic parameters leading to enhanced LiPS conversion under lean electrolyte and high sulfur loading conditions [128]. Although not strictly REE‐based, this work underscores the importance of electronic descriptor control, which is equally applicable to REE‐containing systems. High‐entropy engineering introduces an additional dimension of tunability.

Dual‐site catalyst design has emerged as one of the most effective strategies for decoupling adsorption and conversion functions. Hou et al. (2025) developed Co/Ce dual single‐atom catalysts, where Ce sites preferentially adsorb long‐chain polysulfides while Co sites catalyze Li_2_S nucleation and decomposition [129]. This division of roles enables simultaneous control over intermediate confinement and reaction kinetics, addressing one of the fundamental trade‐offs in Li–S cathode design. Single‐atom REE catalysts anchored on conductive substrates further highlight the importance of atomic dispersion and interfacial engineering. Jing et al. (2025) reported a MXene@CeSA system in which atomically dispersed Ce sites provide abundant catalytic centers, while the MXene substrate ensures efficient electron transport [130]. Such architectures demonstrate how conductive integration and atomic‐level control can synergistically enhance performance, particularly under demanding conditions such as high sulfur loading and lean electrolyte operation.

**FIGURE 11:**
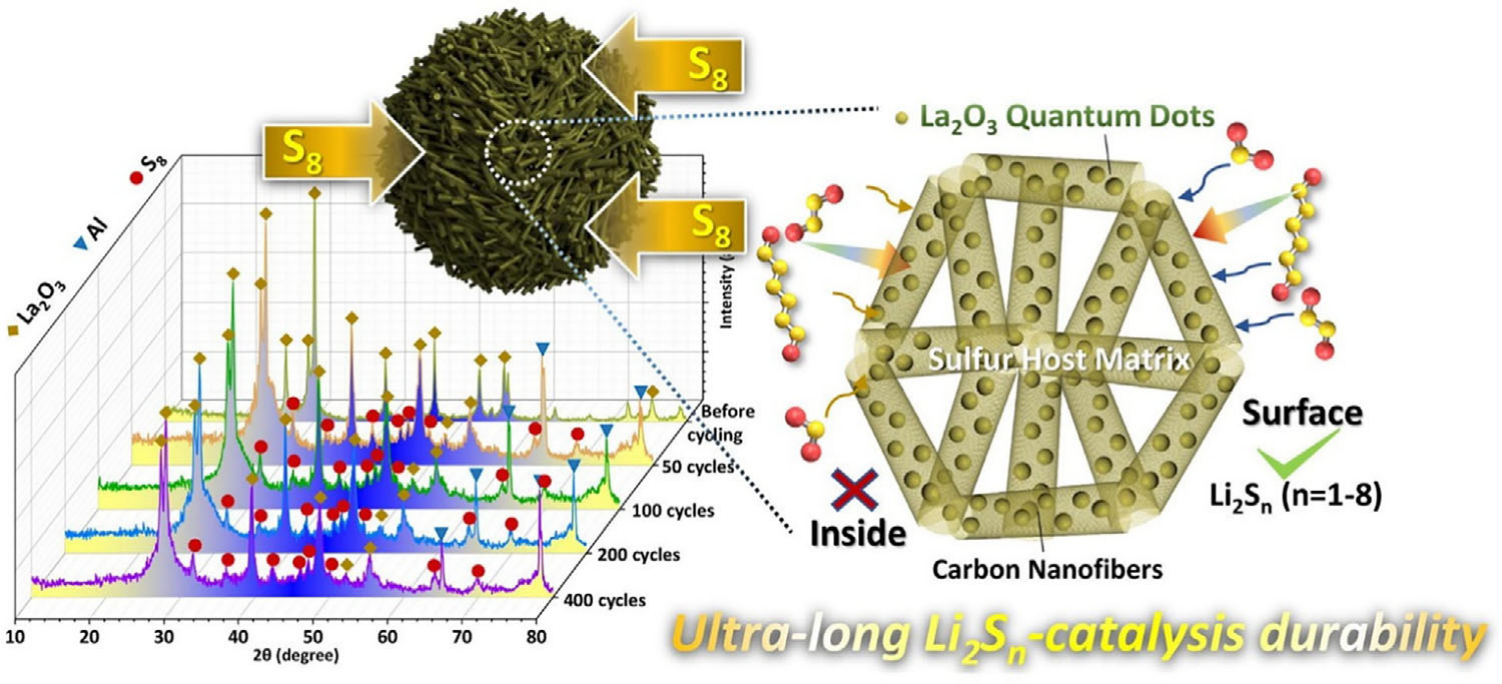
Hybrid sulfur host design based on La_2_O_3_ quantum dots embedded in porous carbon microspheres. Schematic illustration of the composite structure showing uniform confinement of La_2_O_3_ quantum dots within a conductive carbon framework, enabling simultaneous polysulfide adsorption and electron transport. Electron microscopy images before and after extended cycling (50, 100, 200, and 400 cycles) demonstrate the structural stability of the composite and the preservation of active sites [132]. Adapted with permission from Ref. [132]. Copyright 2023 Elsevier.

Hybrid host designs combining REE compounds with carbon frameworks also remain highly effective. Liu et al. (2024) developed LYH@CNT composites, where layered yttrium hydroxide nanosheets provide strong polysulfide adsorption and catalytic conversion, while the CNT network ensures rapid electron transport and structural stability [131]. Similarly, Dai et al. (2023) introduced La_2_O_3_ quantum dots embedded within porous carbon microspheres, where the carbon matrix protects active sites from deactivation while maintaining catalytic activity. Figure 11 illustrates the structural design and working principle of this hybrid host system.

The schematic representation shows that La_2_O_3_ quantum dots are uniformly confined within the porous carbon framework, enabling dual functionality: the REE component provides strong chemical anchoring and catalytic activity, while the carbon matrix offers conductive pathways and physical confinement. Electron microscopy images acquired after extended cycling (50–400 cycles) reveal that the composite maintains its structural integrity, with no significant aggregation or degradation of the active components. These observations highlight a key design principle: combining REE active sites with conductive carbon matrices enables dual confinement chemical adsorption and physical encapsulation while simultaneously ensuring electronic conductivity and structural stability. Such hybrid architectures effectively mitigate catalyst deactivation and polysulfide loss, making them particularly promising for long‐term Li–S battery operation [132].

### 5.2 REE in Separator and Interlayers

The separator and interlayer region plays a decisive role in Li–S batteries by regulating polysulfide transport between the cathode and anode. Unlike cathode hosts, which must balance adsorption and catalytic conversion, separator‐based materials operate as dynamic interfacial regulators, where the primary objective is to intercept, transform, and redistribute migrating polysulfides without impeding Li^+^ transport. In this context, REEs are particularly effective due to their strong Lewis acidity, polar surfaces, and tunable electronic structures. A foundational strategy for advanced Li–S batteries lies in the design of adsorption–conversion coupled interfaces, which aim to mitigate the polysulfide shuttle effect by not only trapping soluble LiPSs but also catalytically accelerating their subsequent transformation, thereby preventing electrode passivation. This principle was effectively demonstrated by Jin et al. [133] through the development of a mixed‐valence cerium‐based metal–organic framework (CSUST‐1), which inherently features Ce^3+^/Ce^4+^ redox couples and abundant oxygen vacancies. When integrated with carbon nanotubes (CNTs), the resulting composite separator functionalization leverages the open cerium sites for strong chemical anchoring of LiPSs, while the Ce^3+^/Ce^4+^ mediation actively promotes the redox kinetics of the trapped species. Furthermore, the porous architecture of the MOF facilitates enhanced lithium‐ion transport. This synergistic combination of strong adsorption and efficient catalytic conversion enabled the battery to achieve exceptional long‐term cycling stability, maintaining performance over 1200 cycles with minimal capacity decay, even under conditions of high sulfur loading. This work underscores how the meticulous coupling of chemical confinement and catalytic activity at the separator can decisively suppress the shuttle effect without compromising the critical reaction kinetics of the sulfur cathode.

Another emerging strategy in the development of advanced Li–S batteries involves the use of dual‐active‐site catalytic separators, where rare earth elements are strategically combined with transition metals to decouple and optimize distinct functionalities. Hong et al. [134] exemplified this approach by introducing a perovskite‐based LaNiO_3_ coating on a polypropylene separator (LaNiO_3_@PP), which leverages the unique roles of its constituent elements. Within this structure, the lanthanum A‐site provides strong polysulfide adsorption through its Lewis acidity, effectively confining the soluble intermediates, while the nickel B‐site serves as an active catalyst to accelerate the conversion of these trapped species. This clear division of labor enables simultaneous and independent control over both intermediate confinement and reaction kinetics, leading to a marked reduction in self‐discharge and improved cycling stability, even under demanding standard and lean‐electrolyte conditions. Such systems compellingly demonstrate that separator coatings can transcend their traditional role as passive physical barriers, evolving instead into sophisticated, active catalytic interfaces that directly participate in and enhance the electrochemical processes of the cell.

A critical challenge in harnessing rare earth compounds for Li–S batteries lies in their intrinsically low electrical conductivity, a limitation that necessitates strategic integration with conductive frameworks to unlock their full electrochemical potential. Addressing this issue, Qian et al. [135] developed a hierarchically structured Y‐MOF‐derived Y_2_O_3_–C@CNT composite, which exemplifies how rational materials design can simultaneously optimize multiple functional requirements. In this architecture, the metal–organic framework serves as a precursor, yielding Y_2_O_3_ nanoparticles embedded within a porous carbon matrix that provides abundant adsorption sites for lithium polysulfides. The concurrent carbonization during synthesis enhances the overall electrical conductivity, while the incorporation of a CNT framework establishes efficient, long‐range electron pathways throughout the electrode structure. This synergistic design enables effective polysulfide trapping coupled with catalytic conversion, all while maintaining robust structural integrity even under demanding high‐sulfur‐loading conditions. The results underscore the importance of hierarchical engineering. When morphology, conductivity, and active site distribution are simultaneously optimized, the resulting composite can transcend inherent material limitations and deliver enhanced electrochemical performance.

Beyond their established roles in adsorption and catalysis, rare earth‐based separator systems demonstrate a more sophisticated capability in regulating local ion transport and interfacial reactions, thereby influencing electrochemical behavior across multiple cell components. The presence of polar rare earth sites on the separator surface can critically modulate lithium‐ion flux distribution and local electric fields within the electrolyte phase, creating more uniform ion concentration gradients that indirectly affect lithium deposition morphology at the anode. This regulatory function becomes increasingly vital under practical operating conditions, where heterogeneous ion flux often exacerbates dendritic lithium growth and accelerates cell degradation. Interestingly, this interfacial regulation blurs the conventional boundary between separator and electrolyte functionality, as demonstrated by Huang et al. [136], who showed that Ce(NO_3_)_3_ can serve as a multifunctional electrolyte additive that participates directly in forming a stable solid electrolyte interphase (SEI) on the lithium metal surface. This Ce‐mediated interphase effectively suppresses dendrite proliferation and enhances cycling stability, illustrating how rare earth elements can act as versatile interfacial regulators across multiple battery components. Although implemented here as an electrolyte additive, the underlying mechanism reflects a broader principle: rare earth species, whether incorporated into separator coatings or dissolved in the electrolyte, can orchestrate interfacial chemistry at both cathode and anode interfaces, suggesting that future separator designs might deliberately harness these dual‐facing regulatory effects to achieve simultaneous suppression of polysulfide shuttling and stabilization of lithium deposition.

From these studies, several important design principles emerge for the development of rare‐earth‐based separator and interlayer systems in lithium–sulfur batteries, collectively pointing toward a paradigm shift in how separator functionality is conceived. First, effective systems must achieve dynamic interception, capturing migrating polysulfides without impeding lithium‐ion transport, thereby maintaining electrochemical kinetics while preventing active material loss. Second, an optimal adsorption‐conversion balance is essential to avoid the passive accumulation of lithium polysulfides on the separator surface, which would otherwise lead to interfacial passivation and capacity decay. Third, conductive integration remains a fundamental requirement, as the catalytic activity of rare earth sites can only be realized when efficient electron transfer pathways are established throughout the separator architecture. Fourth, the concept of dual‐site functionality has proven particularly powerful, wherein distinct components such as rare earth elements for adsorption and transition metals or defect sites for catalysis can be strategically paired to decouple and independently optimize these complementary roles. Fifth and more subtly, interfacial field regulation emerges as a critical design consideration, whereby the polar nature of rare earth species influences local ion flux and reaction distribution, indirectly stabilizing lithium deposition at the anode. Unlike conventional separator modifications that rely primarily on physical blocking or simple sieving effects, these REE‐based systems introduce sophisticated chemical and catalytic control over polysulfide transport, effectively transforming the separator from a passive barrier into an active reaction interface that participates directly in the electrochemical dynamics of the cell.

Despite significant progress, most reported systems are still evaluated under moderate sulfur loadings and excess electrolyte conditions. The effectiveness of REE‐based separator designs under practical conditions (high loading, lean electrolyte, pouch cells) remains insufficiently explored. Furthermore, the interplay between separator chemistry and anode behavior, particularly regarding Li deposition and SEI formation, requires deeper mechanistic investigation. Future research should therefore focus on coupled interface engineering, where separator, electrolyte, and anode modifications are designed in a coordinated manner. In this framework, REEs offer a unique advantage as multifunctional elements capable of bridging these interfaces and enabling system‐level optimization of Li–S batteries.

Integrated, multifunctional deployment of REEs across multiple cell components is conceptually appealing but experimentally underexplored. To date, only a handful of studies have combined, for example, a REE‐based cathode host with a REE‐modified separator and a REE electrolyte additive. The critical question whether such “all‐REE” cells exhibit synergistic benefits or simply additive effects remains unanswered. Moreover, the optimal distribution of REE mass across components (e.g., 80% in cathode, 15% in separator, 5% in electrolyte) has not been systematically optimized. This represents a major knowledge gap because the mechanisms of action (adsorption vs. catalysis vs. ion regulation) differ by component, and trade‐offs are inevitable. Future work should adopt factorial design experiments to deconstruct these interactions.

## 6 Sulfur Loading and Areal Capacity Analysis

The descriptor framework proposed in Section 2 and refined in Section 7 is not an abstract exercise; it directly informs the practical performance metrics discussed here. For example, REEs with moderate crystal‐field splitting and optimal Lewis acidity (e.g., Sm, Gd) are predicted to balance adsorption and conversion, leading to stable cycling under high sulfur loading. This section tests that prediction by benchmarking reported systems against practical metrics.

Achieving high sulfur loading (≥5 mg cm^−2^) and corresponding areal capacity (>4–5 mAh cm^−2^) is essential for translating lithium–sulfur (Li–S) batteries from laboratory‐scale demonstrations to practical applications. While many studies report high specific capacities, these values are often obtained under low sulfur loading (<2 mg cm^−2^) and excess electrolyte conditions, which significantly overestimate real‐world performance. Therefore, evaluation under thick‐electrode conditions provides a more meaningful benchmark for assessing the practical viability of REE‐based systems. Several REE‐enabled architectures have begun to address this requirement. Separator‐based strategies, in particular, show promising scalability. For example, Gd_2_O_3_@C/KB‐modified separators maintained stable cycling performance when sulfur loading increased from 3 to 5 mg cm^−2^, indicating that REE‐based interfacial regulation remains effective even under increased polysulfide flux [137]. Although the electrolyte‐to‐sulfur (E/S) ratio was not reported, this result suggests that REE‐modified separators can sustain electrochemical stability in thick electrodes.

More direct evidence of practical relevance is provided by systems reporting explicit areal capacity. La_2_O_3_/KB@S cathodes achieved 4.9 mAh cm^−2^ at 5 mg cm^−2^ sulfur loading, retaining 3.87 mAh cm^−2^ after 150 cycles [138]. This level of areal performance approaches the threshold required for commercial applications, demonstrating that REE oxides can effectively balance adsorption and catalytic conversion in high‐loading configurations. Similarly, Y_2_O_3_‐based separator systems exhibited strong performance at 5 mg cm^−2^, delivering 831.4 mAh g^−1^ with high retention [139]. Although areal capacity was not explicitly reported, the calculated value (∼4.15 mAh cm^−2^) indicates that such systems are approaching practical benchmarks. Comparable trends are observed in GdS@C‐modified separators, where initial areal capacity (∼4.54 mAh cm^−2^) and stable cycling further confirm the effectiveness of REE sulfide phases under realistic loading conditions [140].

More demanding conditions have also been explored. The Ce–V_2_O_5_/MBene composite, tested at 5.79 mg cm^−2^, maintained stable cycling performance despite significantly increased electrode thickness and polysulfide flux [141]. Although areal capacity was not explicitly reported, the ability to sustain electrochemical activity under such conditions highlights the robustness of REE‐containing hybrid systems. Despite these advances, a critical issue remains: incomplete reporting of key practical metrics. Many studies including CeO_2_‐modified separators demonstrate excellent rate capability and long‐term cycling but omit sulfur loading, areal capacity, or E/S ratio [142]. This lack of standardized reporting severely limits cross‐comparison and obscures true progress toward commercialization.

**FIGURE 12:**
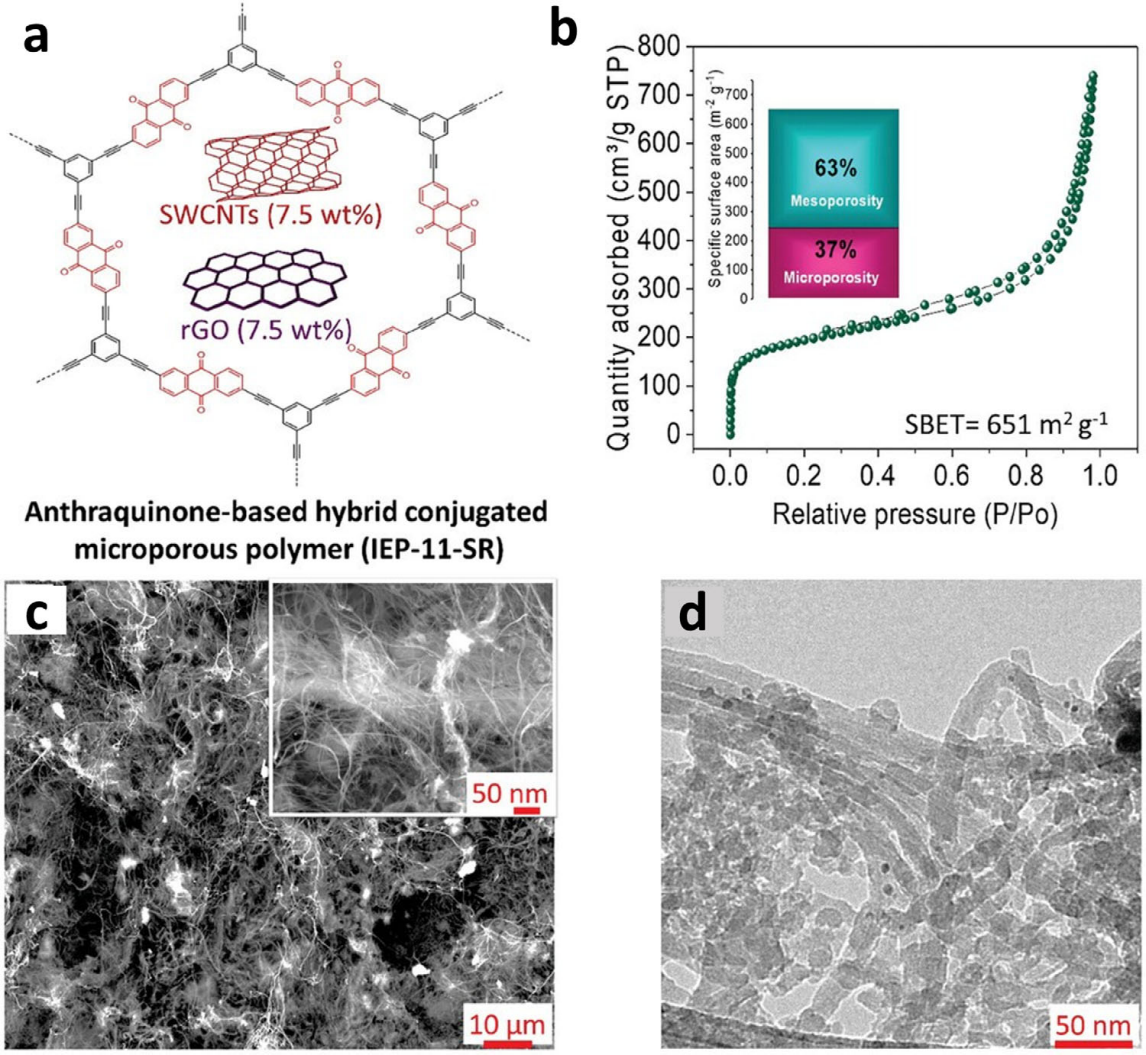
Structural characteristics of a high‐performance polymer electrode (IEP‐11‐SR) enabling thick electrode operation in sodium metal batteries. (a) Schematic of the anthraquinone‐based hybrid conjugated microporous polymer integrated with SWCNTs and rGO, forming a conductive and redox‐active framework. (b) N_2_ adsorption–desorption isotherm and pore size distribution, showing hierarchical porosity with significant micro‐ and mesoporous contributions and high specific surface area. (c) SEM image revealing a porous, interconnected network structure composed of carbon nanostructures. (d) TEM image showing nanoscale morphology and internal porous architecture of the composite. Adapted with permission [143]. Adapted with permission from Ref. [143]. Copyright 2024 Wiley.

To contextualize these results, it is instructive to compare Li–S systems with benchmarks from related battery chemistries. For example, thick electrode engineering in sodium metal batteries has demonstrated areal capacities approaching 7 mAh cm^−2^ at extremely high loadings (≈60 mg cm^−2^) [143]. The material design presented in Figure 12a consists of an anthraquinone‐based conjugated microporous polymer integrated with conductive carbon components (SWCNTs and rGO), forming a hybrid framework that combines redox activity with enhanced electrical conductivity. Figure 12b illustrates the porosity analysis, which reveals a hierarchical structure with a high specific surface area and a balanced distribution of micro‐ and mesopores, supporting both ion storage and efficient electrolyte transport. Furthermore, Figure 12c, the electron microscopy confirms this architecture, which resprent a highly porous, interconnected network composed of entangled carbon nanotubes and graphene sheets, creating continuous electron transport pathways and accessible pore channels. At higher resolution, TEM analysis Figure 12d reveals the nanoscale internal structure, highlighting the presence of well‐developed porous domains and intimate integration between the polymer matrix and conductive components. These combined structural features establish a key design principle: hierarchical porosity coupled with an interconnected conductive network enables high mass loading while maintaining efficient ion and electron transport. While Li–S systems face additional challenges such as polysulfide shuttling, this benchmark underscores the importance of multi‐scale structural engineering and highlights the gap between current sulfur cathodes and the requirements for practical, high‐loading battery systems.

Cost‐effective and scalable material strategies further reinforce the importance of practical metrics. The ESrp@CoP separator, based on naturally abundant serpentine substrates, achieved stable cycling at 3.9 mg cm^−2^ with good retention [144]. Although slightly below the 5 mg cm^−2^ benchmark, such systems demonstrate that scalability and cost considerations must be evaluated alongside electrochemical performance. Finally, volumetric capacity remains an underreported but critical parameter. Ma et al. demonstrated that densified electrode architectures can achieve high volumetric capacities while maintaining areal scalability, emphasizing the importance of electrode density and thickness optimization [145]. This perspective is particularly relevant for REE‐based systems, where porous structures often sacrifice volumetric energy density for improved kinetics.

These studies demonstrate that rare earth‐based materials possess the intrinsic functionality to sustain high sulfur loading and approach practical areal capacity targets, underscoring their potential for real‐world Li–S battery applications. However, a critical examination of the literature reveals that progress remains uneven and the true commercial relevance of many reported systems is difficult to assess due to several persistent shortcomings in experimental evaluation. Inconsistent reporting of electrolyte‐to‐sulfur (E/S) ratio and areal capacity across studies undermines meaningful comparison, as these parameters directly influence both the observed performance and the projected viability in practical cells. Furthermore, limited evaluation under lean‐electrolyte conditions a non‐negotiable requirement for achieving high energy density means that many materials have not been tested under the rigorous conditions they would face in commercial applications.

Compounding this issue is the widespread absence of volumetric energy density analysis, which is particularly critical for rare earth‐based systems where the incorporation of dense elements may compromise the volumetric advantages essential for portable and vehicular energy storage. Finally, insufficient testing in pouch‐cell configurations, as opposed to coin cells, leaves unanswered questions regarding scalability, mechanical robustness, and failure modes under realistic cell formats and pressures. Consequently, while the fundamental advances in adsorption‐conversion coupling, dual‐site functionality, and interfacial regulation are undeniably promising, the field must now pivot toward more rigorous and standardized evaluation protocols that prioritize commercially relevant metrics, ensuring that the next generation of reported “high‐performance” systems can be assessed not only by their laboratory achievements but by their demonstrable potential to meet the demanding requirements of practical lithium–sulfur batteries.

This assessment correctly identifies the critical juncture at which the field of rare earth‐based Li–S batteries now stands. Future work must indeed transcend the prevailing focus on demonstrating high specific capacity in idealized laboratory settings and instead pivot toward establishing standardized evaluation protocols that reflect the demands of practical energy storage. This necessitates rigorous testing under conditions that include sulfur loadings of at least 5–10 mg cm^−2^, electrolyte‐to‐sulfur ratios no greater than 5 µL mg^−1^, and areal capacities meeting or exceeding 5 mAh cm^−2^, all while ensuring consistent reporting of both gravimetric and volumetric performance metrics to enable meaningful cross‐study comparison and technoeconomic assessment.

Within this more demanding framework, rare earth‐based materials continue to show clear promise, particularly when deployed in separator and hybrid architectures where their unique adsorption‐conversion coupling, dual‐site functionality, and interfacial regulatory capabilities can be most effectively harnessed. However, their ultimate success will be determined not solely by their catalytic prowess or polysulfide affinity as measured in coin cells, but by their demonstrated ability to operate reliably, safely, and consistently under the industrially relevant conditions that govern real‐world battery manufacturing and deployment. The transition from materials innovation to engineering validation thus represents the next frontier, requiring the community to embrace evaluation standards that are as rigorous as the chemistry itself.

### 6.1 Performance Under Practical Conditions

Except for sulfur loading and areal capacity, practical Li–S batteries must demonstrate long‐term cycling stability, high‐rate capability, and device‐level validation. These criteria are particularly important because many materials that perform well under mild conditions fail when subjected to high current densities or extended cycling. In this context, REE‐based systems must be evaluated not in isolation, but against state‐of‐the‐art transition‐metal and carbon‐based benchmarks. REE‐based separator and interlayer strategies have shown promising performance under demanding conditions. For example, Peng et al. developed an Eu_2_O_3_/KB‐coated separator that delivered 563 mAh g^−1^ at 3C and maintained a low capacity decay of 0.05% per cycle over 500 cycles at 1C [146]. This performance highlights the effectiveness of combining Lewis acidic adsorption with oxygen‐vacancy‐assisted catalytic conversion, suggesting that REE oxides can provide an alternative mechanistic pathway distinct from traditional d‐band‐driven catalysis. Among REE systems, CeVO_4_/KB represents a particularly strong benchmark, achieving a decay rate of only 0.063% per cycle over 1000 cycles at 3C while maintaining high initial capacity [147]. Such results demonstrate that REE‐based materials can sustain both high‐rate operation and long‐term durability, two requirements often considered difficult to achieve simultaneously.

However, when compared to optimized non‐REE systems, a performance gap remains. For instance, indium‐derived porous carbon microspheres (PCMS) achieved extremely low decay rates of 0.014% per cycle over 500 cycles at 4C and 0.060% over 900 cycles at 2C, even at relatively high sulfur loading [148]. Similarly, transition‐metal‐based separator systems such as CoFe@g‐C_3_N_4_ exhibit strong high‐rate performance and cycling stability, establishing a competitive baseline for evaluating REE‐based designs [149]. At the extreme end, durability benchmarks from related systems further emphasize the challenge. For example, C/CoFe‐MXene sulfur hosts in Na–S batteries demonstrated over 5400 cycles with ultralow decay (0.0089% per cycle), highlighting the level of stability achievable when conductivity, adsorption, and catalytic activity are fully optimized [150].

Mid‐range systems provide additional context for realistic evaluation. ZRNO@C‐modified separators, for example, delivered moderate cycling stability (∼0.11% decay per cycle over 500 cycles), offering a useful reference point for interpreting exceptionally low decay values reported in high‐performance systems [151]. Likewise, multifunctional separator designs such as Janus‐type architectures demonstrate that combining catalytic layers with anode protection can significantly enhance overall cell durability [152]. These comparisons highlight an important insight: ultralow capacity fade alone is not sufficient to claim superiority. Instead, performance must be evaluated in the context of sulfur loading, E/S ratio, and structural complexity.

A critical step toward commercialization is validation beyond coin cells. In this regard, pouch‐cell demonstrations remain scarce for REE‐based Li–S systems. One notable example is the MXene@MMT interlayer, which maintained stable performance when translated from coin cells to soft‐pack batteries, delivering 733 mAh g^−1^ initially and retaining 402 mAh g^−1^ after 100 cycles [153]. Although the cycle number remains limited, such demonstrations significantly enhance the practical credibility of the design. Benchmark comparisons from other battery systems further illustrate the required performance envelope. For instance, high‐loading LiCoO_2_‐based cells achieve stable cycling with areal capacities of ∼2.8 mAh cm^−2^ over hundreds of cycles [154], while solid‐state Al–S pouch cells have demonstrated >90 Wh kg^−1^ energy density with extended cycling stability [155]. These results provide a device‐level reference framework that Li–S systems must approach to be considered commercially viable.

An often‐overlooked factor in separator and interlayer design is the mass and density penalty. Zou et al. addressed this issue by developing a lightweight CeF_3_/CNT interlayer with ultralow density, demonstrating that interlayer design must balance functionality with minimal weight contribution [156]. This perspective is critical, as excessive interlayer mass can negate gains in gravimetric energy density. A broader comparison with non‐rare‐earth systems reveals that similar or even superior electrochemical performance can be achieved through optimized material design polyaniline/reduced graphene oxide cathodes demonstrate ultralow capacity decay, advanced carbon architectures deliver excellent rate stability, and transition‐metal catalysts provide strong polysulfide conversion activity. These observations raise an important question: are rare earth elements uniquely enabling for Li–S batteries, or simply one of several viable pathways? Current evidence suggests that while rare earths offer distinct advantages in terms of polarity, redox flexibility, and defect chemistry, their superiority over well‐engineered alternatives is not yet universally established. Their true value may therefore lie less in outperforming all competitors on any single metric, and more in their capacity to enable multifunctional and interface‐spanning designs that simultaneously address challenges across multiple cell components.

Across all systems, the most critical determinant of practical performance is not a single material property, but the integration of adsorption, catalysis, conductivity, and interfacial stability within a unified architecture. REE‐based materials are particularly well‐suited for this role, but their effectiveness depends on how well they are integrated into the overall cell design.

### 6.2 Cost, Abundance, and Criticality of REEs

While REEs offer unique catalytic and interfacial advantages in Li–S batteries, their large‐scale deployment is ultimately constrained by cost, abundance, and supply‐chain stability. Unlike sulfur, which is abundant and inexpensive REEs, particularly mid‐ and heavy‐lanthanides, are associated with limited availability, geographically concentrated production, and environmentally intensive extraction processes. As a result, evaluating REE‐based battery technologies requires not only electrochemical performance metrics but also consideration of resource sustainability and economic feasibility. A key distinction must be made between light REEs (La, Ce, Nd) and heavy REEs (Gd, Tb, Dy, Yb). Light lanthanides are relatively more abundant and widely used in industrial applications, making them more viable for large‐scale deployment.

In contrast, heavy REEs are significantly scarcer and often co‐produced in limited quantities, leading to higher costs and supply risks. This disparity has direct implications for material design. Many high‐performance systems rely on elements such as Gd or Yb, which may not be sustainable at scale. Consequently, future REE‐based strategies must prioritize either earth‐abundant REEs (e.g., Ce, La) or ultralow loading / single‐atom utilization to minimize material demand without sacrificing functionality.

To address supply constraints, recent studies have explored REE recovery and recycling pathways, which are essential for establishing circular material flows. Liao et al. demonstrated a graphene oxide‐based aerogel capable of efficient REE adsorption with rapid equilibration and excellent regeneration stability [157]. Notably, the system maintained near‐complete adsorption capacity and high desorption efficiency over multiple cycles, highlighting the feasibility of reusable REE capture systems. Similarly, Engmann et al. reported pseudocapacitive adsorption of Nd^3+^ on carbon electrodes, achieving high selectivity and retention over repeated cycles [158]. Importantly, these results emphasize that selective recovery from complex mixtures, rather than simple adsorption capacity, is critical for practical recycling systems. In real‐world waste streams, the ability to distinguish REEs from competing ions (e.g., Li^+^, Mg^2+^) will determine the viability of recovery technologies.

**FIGURE 13:**
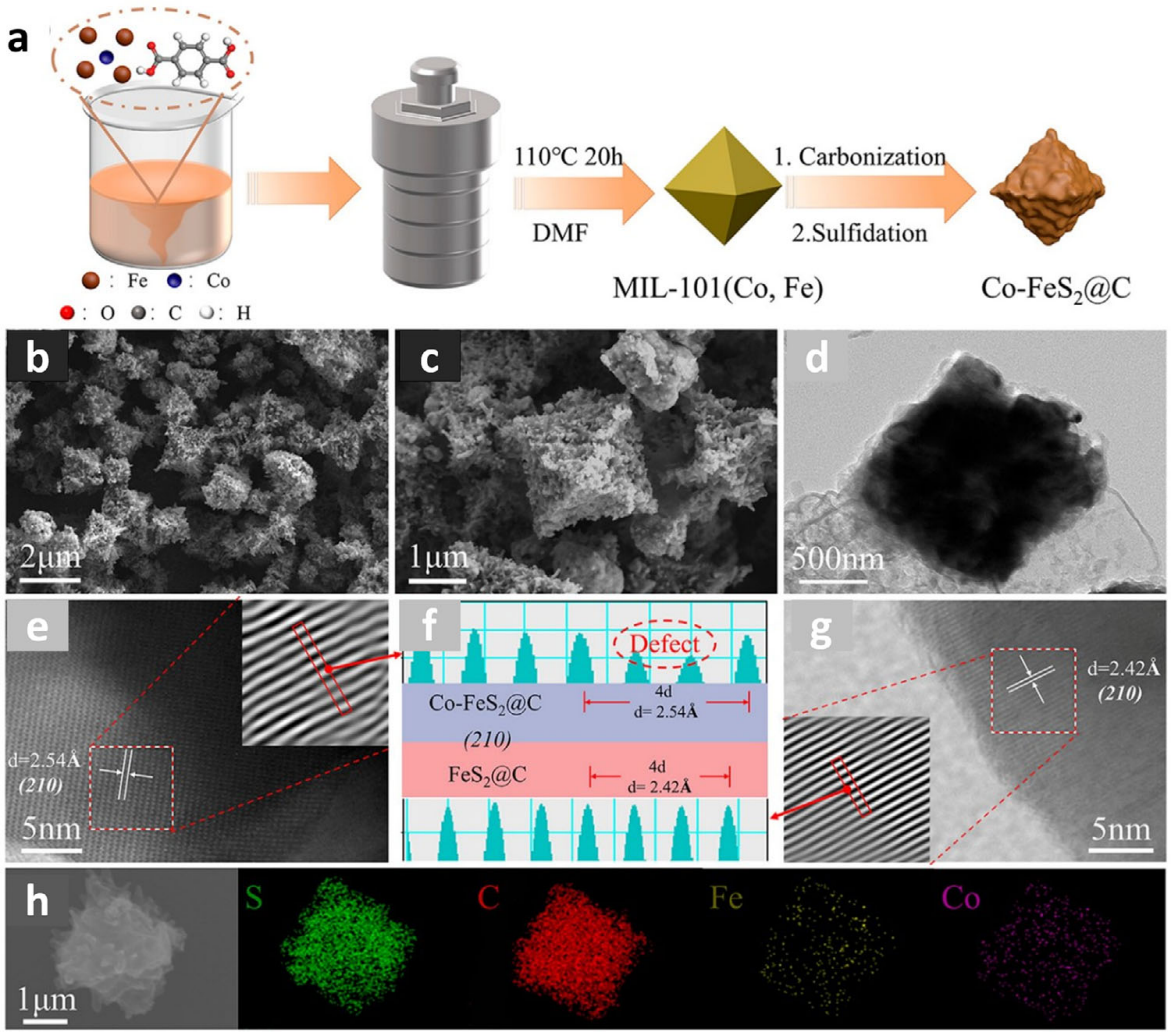
Structural and morphological characterization of Co–FeS_2_@C composites for rare‐earth ion recovery. (a) Synthesis schematic showing conversion of bimetallic MIL‐101(Co, Fe) into Co–FeS_2_ nanoparticles embedded in a porous carbon matrix via carbonization and sulfidation. (b, c) SEM images at different magnifications reveal porous, aggregated particle morphology. (d) TEM image showing the nanoscale structure of the composite. (e, g) HRTEM images displaying lattice fringes corresponding to Co–FeS_2_, with interplanar spacings indicating crystal structure and lattice modulation due to Co incorporation. (f) Intensity profile analysis highlighting lattice variation and defect formation. (h) Elemental mapping showing uniform distribution of S, C, Fe, and Co within the composite [159]. Adapted with permission from Ref. [159]. Copyright 2025 MDPI.

More advanced material systems have been developed to improve both capacity and stability for REE recovery. For example, Bi et al. designed a Co–FeS_2_@C composite for Yb^3+^ electrosorption, achieving high adsorption capacity (129.2 mg g^−1^) and excellent cycling stability [159]. Figure 13 illustrates the structural design and multi‐scale characterization of this composite, highlighting how its architecture enables efficient and stable ion capture. The synthesis strategy in Figure 13a involves the transformation of a bimetallic MOF precursor (MIL‐101(Co, Fe)) into a carbon‐encapsulated sulfide composite, ensuring uniform dispersion of active sites within a conductive matrix. The morphological analysis in Figures 13b–d shows that the material consists of porous, micron‐scale particles with embedded nanoparticles, while elemental mapping confirms homogeneous distribution of Co and Fe without phase segregation. This structural uniformity is essential for consistent adsorption performance. Figure 13e–g shows that at the nanoscale, high‐resolution imaging reveals well‐defined lattice fringes corresponding to Co–FeS_2_, along with measurable variations in interplanar spacing compared to FeS_2_‐only systems. These differences indicate that Co incorporation induces lattice distortion and defect formation. The presence of such defects is further supported by intensity profile analysis in Figure 13f, which suggests local structural irregularities that can serve as additional active sites for ion interaction. Furthermore, the elemental mapping in Figure 13h confirms the uniform spatial distribution of S, C, Fe, and Co throughout the composite, demonstrating successful integration of all functional components within the carbon framework. This ensures that active sites are both chemically accessible and electronically connected.

From a battery‐design perspective, the use of rare earth elements introduces a critical trade‐off between performance enhancement and material cost. While rare earths can significantly improve polysulfide regulation and catalytic kinetics, their contribution to overall cell cost must be carefully managed. In this regard, several strategies emerge for maximizing the functional efficiency per unit mass of rare earth element, which is ultimately a more relevant metric than absolute performance. These approaches include minimizing rare earth content through atomic dispersion or ultrathin coating strategies, utilizing rare earths selectively in separators and interlayers where smaller quantities are required, combining rare earth components with low‐cost substrates such as carbon or natural minerals, and targeting multifunctionality so that a single rare earth component can replace multiple distinct materials within the cell architecture.

Despite promising advances in the laboratory, the integration of rare earth elements into Li–S batteries remains at an early stage from a commercialization standpoint. Current studies often overlook several critical factors that would determine real‐world viability, including the actual mass fraction of rare earth elements in the full cell configuration, the cost contribution of these materials relative to the performance gains they enable, the scalability of synthesis methods beyond the gram scale, and the availability of recycling infrastructure to recover rare earth components at the end of life. Moreover, the reliance on heavy rare earth elements in some high‐performance systems raises legitimate concerns about long‐term sustainability and supply chain security, given the geopolitical concentration and environmental footprint associated with heavy rare earth mining and processing.

The viability of REE‐based Li–S technologies will depend not only on electrochemical performance, but on achieving a balance between: material efficiency (low REE usage), functional effectiveness (high catalytic activity), and supply sustainability (recyclability and abundance). Future research should therefore prioritize a strategic realignment of focus, shifting toward light and abundant rare earth elements such as cerium‐ and lanthanum‐based systems that offer greater supply security and lower environmental impact. This must be coupled with the development of closed‐loop recycling strategies that are integrated into battery design from the outset, enabling material recovery and circular economy principles to take hold. Critical to this transition is the quantification of cost‐to‐performance ratios alongside traditional electrochemical metrics, ensuring that performance enhancements are evaluated not in isolation but against their economic and practical implications. Finally, researchers must design rare earth‐efficient architectures that minimize material consumption through atomic dispersion, thin coatings, or targeted interfacial placement, thereby maximizing functional efficiency per unit mass. In this context, rare earth elements should not be viewed as bulk components to be incorporated liberally, but rather as strategically deployed functional elements, where their unique coordination chemistry, redox versatility, and interfacial activity are leveraged with minimal material input to achieve maximum electrochemical impact.

### 6.3 Comparison With Transition‐Metal Catalysts

The catalytic behavior of transition metals and REEs in Li–S batteries arises from fundamentally different electronic origins, leading to distinct reaction pathways, stability characteristics, and design strategies. Transition‐metal catalysts operate through partially filled d‐orbitals, where adsorption strength and reaction kinetics are governed by the position of the d‐band center relative to the Fermi level. This framework enables systematic tuning of polysulfide binding energies; however, overly strong metal–sulfur interactions can result in irreversible sulfide formation, surface passivation, or sluggish desorption during extended cycling. In contrast, REEs possess localized and shielded 4f orbitals, which contribute less directly to covalent bonding. Their catalytic function is instead dominated by: Lewis acidity (strong electrostatic interaction with polysulfide anions), defect chemistry (particularly oxygen vacancies), and multivalent redox couples (e.g., Ce^3+^/Ce^4+^, Eu^2+^/Eu^3+^). As a result, REE‐based materials typically follow an adsorption–conversion mechanism mediated by ionic interactions and surface defects, rather than d‐band‐controlled chemisorption. These electronic differences translate into distinct stability profiles. Transition‐metal sulfides, phosphides, or nitrides often undergo surface reconstruction or chemical poisoning during prolonged exposure to polysulfides, which can alter active‐site structure and reduce catalytic activity over time.

By contrast, REE compounds, particularly oxides and fluorides are generally thermodynamically stable, with strong metal–oxygen bonds and resistance to bulk phase transformation. Their interaction with sulfur species is predominantly surface‐mediated, which contributes to improved structural robustness, reduced susceptibility to irreversible reactions, and more stable long‐term cycling behavior. However, this stability comes at the cost of low intrinsic electronic conductivity, necessitating integration with conductive matrices such as carbon or MXene frameworks.

The performance differential between rare‐earth‐based and transition‐metal‐based catalyst systems becomes particularly evident when examining the practical metrics summarized in Table 5, revealing distinct strengths and current limitations for each material class. Rare earth‐based systems demonstrate stable operation at relatively high sulfur loadings approaching approximately 5 mg cm^−2^, enabling areal capacities in the range of 4–5 mAh cm^−2^ while maintaining long‐term cycling stability extending to hundreds or even thousands of cycles. Specific examples illustrate this capability: La_2_O_3_/KB and GdS@C composites approach the critical 5 mAh cm^−2^ benchmark, while CeVO_4_/KB and Eu_2_O_3_/KB systems exhibit remarkably low capacity decay under high‐rate cycling conditions. These results collectively highlight the strength of rare earth elements in maintaining electrochemical stability under practical operating conditions, particularly when deployed as separator coatings or interlayers where their adsorption and interfacial regulation functions can be most effectively harnessed.

**TABLE 5:** Numerical Benchmark Summary of Practical Metrics.

System	Loading (mg cm^−2^)	Areal (mAh cm^−2^)	Cycles	Decay %/cycle	Rate	Refs.
Gd_2_O_3_@C	5	∼4^+^	500	0.088	0.5C	[137]
La_2_O_3_/KB	5	4.9 → 3.87	150	—	0.3C	[138]
Y_2_O_3_/YS@C	5	∼4.15	100	—	0.1C	[139]
GdS@C	5	∼4.54	100	—	0.1C	[140]
CeVO_4_/KB	—	—	1000	0.063	3C	[147]
Eu_2_O_3_/KB	—	—	500	0.05	3C	[146]
CeF_3_/CNT	—	—	500	0.063	1C	[156]
C/CoFe‐MXene	—	—	5400	0.0089	5C	[150]
PGS/GO	—	—	500	0.02	1.67C	[160]

In contrast, transition‐metal‐based systems such as C/CoFe‐MXene composites demonstrate exceptional cycling durability and rate capability, achieving ultralow decay rates on the order of 0.0089% per cycle over thousands of cycles. This comparative performance indicates that transition‐metal catalysts, when their conductivity and catalytic activity are fully optimized through advanced materials engineering, still define the upper limit of achievable performance in lithium–sulfur batteries, underscoring that rare earth elements currently occupy a complementary rather than dominant position in the catalytic materials landscape.

A fundamental advantage enjoyed by transition‐metal catalysis lies in the availability of a well‐defined theoretical descriptor the d‐band center, which enables predictive catalyst design through established correlations between electronic structure and adsorption energetics. In comparison, rare‐earth‐based systems currently lack a unified descriptor framework, representing a significant gap in the fundamental understanding required for rational materials optimization. Emerging studies suggest that the catalytic behavior of rare earth elements may be governed by a more complex interplay of multiple parameters, including oxygen vacancy concentration, Lewis acidity strength, redox accessibility through accessible oxidation state pairs such as Ce^3+^/Ce^4+^ or Eu^2+^/Eu^3+^, and defect formation energy coupled with surface electrostatics.

Developing a quantitative descriptor that integrates these interdependent factors represents a critical step toward establishing a predictive design framework for rare earth catalysts, analogous to the role that d‐band theory has played in advancing transition‐metal catalysis. Such a descriptor would not only accelerate the identification of high‐performance compositions but also enable meaningful cross‐system comparisons and provide fundamental insights into the unique catalytic mechanisms that distinguish rare earth elements from their transition‐metal counterparts. Rather than positioning rare earth elements as direct competitors to transition‐metal catalysts, a more constructive perspective emerges when they are viewed as representing a complementary catalytic paradigm within Li–S battery design. Transition metals offer high intrinsic activity and strong electronic coupling with sulfur species, enabling rapid reaction kinetics, yet this comes with a risk of instability under cycling conditions and potential passivation over extended operation.

REEs, by contrast, provide high chemical stability, tunable polarity arising from their unique electronic configurations, and defect‐driven catalysis mediated by oxygen vacancies and surface electrostatics, though they remain limited by inherently low electrical conductivity that necessitates conductive integration. This complementary relationship suggests that the most effective systems may arise not from choosing one class over the other, but from deliberately engineered hybrid architectures in which rare earth components provide structural stability and adsorption control while transition metals contribute high catalytic activity, thereby harnessing the distinct strengths of each material class to achieve performance characteristics unattainable by either alone.

Current evidence suggests that rare earth‐based materials are particularly advantageous when deployed in separator and interlayer configurations, where their strong polarity and inherent chemical stability can effectively regulate polysulfide transport and interfacial reactions without imposing a significant mass penalty on the overall cell architecture. In this role, the unique adsorption and defect‐driven properties of rare earth elements are leveraged precisely where they are most needed at the interface between cathode and separator while their conductivity limitations are mitigated through thin coatings or composite integration.

However, for rare‐earth‐based systems to compete directly with transition‐metal catalysts at the cathode level, where electronic participation in redox reactions is paramount, further advances in electronic conductivity, active‐site density, and descriptor‐guided design will be required to approach the kinetic performance of optimized transition‐metal compounds. Ultimately, the future of rare earth‐based lithium–sulfur systems lies not in positioning them as replacements for transition metals, but rather in enabling multifunctional, hybrid, and interface‐engineered architectures that strategically combine the stability and polarity of rare earths with the high catalytic activity of transition metals, thereby achieving performance synergies that neither material class could realize independently.

When directly comparing the best REE‐based systems (e.g., CeVO_4_/KB, Eu_2_O_3_/KB) with optimized transition‐metal catalysts (e.g., C/CoFe‐MXene, CoP_2_), a clear pattern emerges: transition metals generally achieve lower Tafel slopes (by 20–30 mV dec^−1^) and higher rate capabilities (by a factor of 2–3 at 5C), but REEs exhibit superior cycling stability under lean‐electrolyte conditions and better tolerance to polysulfide poisoning. This trade‐off suggests that REEs are not “better” than transition metals; rather, they occupy a complementary niche. A second critical insight is that many claims of REE superiority stem from comparing against poorly optimized transition‐metal controls (e.g., bulk Co_3_O_4_ instead of single‐atom Co). When fair comparisons are made (e.g., Ce single‐atom vs. Co single‐atom on identical carbon supports), the performance gap narrows considerably. Therefore, future studies must include state‐of‐the‐art transition‐metal benchmarks under identical test conditions.

## 7 Outlook: Descriptor‐Guided Design and Future Directions

The central argument of this review is that REE‐based Li–S catalysts cannot be rationally designed using a single descriptor analogous to the d‐band center. Instead, they require a multi‐parameter descriptor framework that captures the unique physics of 4f orbitals. This framework comprises five interdependent descriptors: (i) crystal‐field splitting (ΔCF), (ii) electronegativity (χ), (iii) f–d hybridization strength (Hfd), (iv) oxygen vacancy concentration (δ), and (v) Lewis acidity (pKa of the REE aqua ion). The following sections outline how these descriptors collectively govern adsorption energetics, Li_2_S nucleation barriers, and catalytic turnover, and how they can be integrated into a predictive, data‐driven design pipeline.

REEs have emerged as a distinctive class of functional materials for Li–S batteries, offering catalytic behavior fundamentally different from conventional transition‐metal systems. Their localized 4f orbitals, variable oxidation states, strong Lewis acidity, and defect chemistry enable unique adsorption–conversion mechanisms that are particularly effective for regulating polysulfide chemistry. However, the field is now at a critical transition point: further progress requires moving beyond empirical material discovery toward predictive, mechanism‐driven design. A central challenge lies in the absence of a unified descriptor analogous to the d‐band center that governs transition‐metal catalysis.

Recent advances in data‐driven and machine‐learning‐assisted approaches have begun to address this gap in related catalytic systems, providing a blueprint for REEs. For example, a collaborative data‐driven model analyzing over 2900 articles on the sulfur reduction reaction identified a composite descriptor that successfully predicted the catalytic activity of over 800 types of catalysts and enabled the discovery of tens of novel SRR catalysts from 374 833 candidates [161]. Similarly, systematic descriptor‐based research paradigms have delineated electronic, structural, and energy descriptors, highlighting how emerging AI methodologies can facilitate the further development of reactivity descriptors for Li–S batteries [162]. Furthermore, a multi‐view machine‐learned framework has demonstrated that orbital coupling among active sites induces shifts in band centers and alterations in spin state, directly influencing polysulfide interactions and Li–S bond breaking concepts directly transferable to REE‐based systems where 4f orbitals play an analogous role [163].

Current evidence indicates that REE‐based systems are controlled by a multi‐parameter framework in which catalytic performance arises from the interplay of several interdependent descriptors. Key factors include crystal‐field splitting, which governs electronic structure and adsorption energetics; electronegativity, which modulates bond covalency and polysulfide binding strength; f–d hybridization, which enables synergistic activity in hybrid systems; and oxygen vacancy concentration, which defines active sites and redox reactivity.

In parallel, Lewis acidity provides a chemically intuitive descriptor, as REE–polysulfide interactions are largely driven by electrostatic attraction and coordination chemistry. The integration of these parameters into a quantitative descriptor framework represents a critical step toward rational catalyst design and remains one of the most important unresolved challenges in the field.

Machine learning offers a powerful pathway to integrate these multi‐dimensional descriptors. DFT and ML insights on the orbital dependence of single‐metal sites have successfully identified key features governing Li_2_S binding strength, demonstrating that ML models can predict adsorption energy and reveal the key substrate features influencing binding [164]. Moreover, the emerging integration of density functional theory with machine learning, especially interpretable ML models, has shown great promise for accelerated catalyst screening and deeper mechanistic understanding [165].

An explainable AI‑driven approach has been specifically developed to intelligently design catalysts adaptive to diverse local chemical environments in Li–S batteries, achieving exceptional catalytic and battery performance under practical lean‑electrolyte conditions [166]. These data‐driven paradigms offer a clear roadmap for REEs: by training ML models on DFT‐calculated adsorption energies, activation barriers, and defect formation energies across the lanthanide series, it will be possible to identify the most promising REE‐based catalyst candidates before any experimental synthesis.

At the same time, the exploration of REEs in Li–S batteries remains highly uneven. Current research is dominated by cerium‐ and lanthanum‐based systems, while the majority of the lanthanide series remains underexplored. Mid‐lanthanides such as Sm and Gd have already demonstrated promising catalytic behavior driven by vacancy chemistry and electronic modulation, suggesting a broader and largely untapped design space. Heavy lanthanides, including Yb and Lu, exhibit favorable electronic properties and strong catalytic potential, particularly in single‐atom configurations, but their limited abundance and higher cost necessitate low‐loading or atomically dispersed strategies.

A systematic investigation of periodic trends across the lanthanide series is therefore essential to establish performance–cost relationships and guide element selection. High‐throughput DFT calculations offer a practical pathway to systematically screen undeveloped lanthanide elements. In the broader computational materials science community, high‐throughput DFT screening of single‐atom catalysts has already been successfully demonstrated across 3d, 4d, and 5d transition metals as well as lanthanides, revealing that defect formation energy is highly correlated with electron affinity differences, electronegativity, and boiling point [167].

For REEs specifically, DFT studies on rare‐earth single‐atom metals supported on C_2_N have systematically evaluated their catalytic performance, demonstrating that the binding energy of adsorbed oxygen atoms can serve as an effective activity descriptor [168]. These studies provide a direct methodological template for extending high‐throughput DFT screening to REE‐based Li–S catalysts, where adsorption energies for key polysulfide intermediates (Li_2_S_4_, Li_2_S_2_, Li_2_S) and activation barriers for Li_2_S nucleation can be computed across the entire lanthanide series under identical conditions. Such an approach would transform the current empirical element selection into a rational, descriptor‐guided process.

A further limitation of current studies is the reliance on indirect or ex situ evidence to infer catalytic mechanisms. Advancing the field requires the adoption of operando and multiscale characterization techniques capable of probing dynamic processes under realistic conditions. Techniques such as operando XAS, Raman spectroscopy, and surface‐sensitive analyses must be integrated with density functional theory and molecular dynamics simulations to establish quantitative structure–function relationships.

This combined experimental–theoretical approach is essential for identifying true active sites, validating descriptor‐based models, and capturing the dynamic evolution of REE catalysts during cycling. MLcan also play a crucial role here: interpretable ML models have already been used to identify the most relevant features dictating Li_2_S binding on catalytic surfaces, and similar approaches can be applied to operando datasets to extract hidden correlations between REE oxidation state dynamics and catalytic activity [164].

Future progress will depend on integrating REEs into complete battery architectures. The most promising strategies involve multifunctional deployment across cathodes, separators, and anodes, where REEs act as interfacial regulators rather than isolated catalysts. In this context, hybrid systems combining REEs with transition metals or conductive frameworks offer a particularly powerful pathway, leveraging the stability and polarity of REEs alongside the high catalytic activity of d‐band systems. Data‐driven approaches have already been successfully applied to design transition‐metal‐based catalysts for high‐energy Li–S pouch cells, achieving an initial specific energy of 436 Wh kg^−1^ under lean‐electrolyte conditions [163]. Extending such methodologies to REE‐based systems, by incorporating 4f‐orbital descriptors (crystal‐field splitting, f–d hybridization, oxygen vacancy concentration) into the feature space, represents a clear and actionable future direction.

Finally, the long‐term viability of REE‐based Li–S technologies must be evaluated within a sustainability and circular economy framework. The limited availability and uneven distribution of certain lanthanides necessitate strategies that minimize material usage, prioritize abundant elements such as Ce and La, and incorporate efficient recycling pathways. Advances in REE recovery through adsorption and electrosorption highlight the feasibility of closed‐loop material systems, but integration of these approaches into battery design remains an open challenge.

Light REEs (La, Ce, Nd) are relatively abundant ( $2–5 kg^−1^ for La_2_O_3_) and can be deployed at scale. Heavy REEs (Gd, Yb, Lu) are scarce ( $30–50 kg^−1^) and should be used only in atomically dispersed form (<0.1 wt.%). Recent examples of effective recovery include graphene oxide aerogels achieving >95% adsorption efficiency over multiple cycles  [157], pseudocapacitive electrosorption with high selectivity for Nd^3^^+^ over Li^+^ and Mg^2+^ [158], and Co–FeS_2_@C composites for Yb^3+^ recovery with a capacity of 129.2 mg g^−1^ and excellent cycling stability [159].

A critical future direction is the integration of recovery functionality directly into battery design, for example, using magnetic REE oxides or water‐soluble REE salts that can be easily leached at the end‐of‐life. In this context, the future of REE‐based Li–S batteries will be defined not by isolated material innovations, but by the convergence of descriptor‐guided design, machine‐learning‐assisted high‐throughput screening, systematic element selection (including the full lanthanide series from La to Lu), advanced operando characterization, and sustainable deployment strategies. By embracing this integrated approach, REEs can evolve from niche catalytic additives into key components of next‐generation energy storage systems.

### 7.1 Proposed Multi‐Descriptor Framework

From our critical survey of the literature, five descriptors emerge as the most influential for REE‐based Li–S catalysts. Crystal‐field splitting (ΔCF) governs the energy gap between 4f orbitals and influences adsorption geometry; larger ΔCF typically weakens covalent bonding but enhances electrostatic interactions. Electronegativity (χ) modulates bond ionicity: lower χ (e.g., La, Ce) favors stronger ionic interactions with polysulfides, while higher χ (e.g., Lu) increases covalency. f–d hybridization strength (Hfd) is critical when REEs are combined with transition metals; stronger Hfd (e.g., Yb, Lu) enhances charge transfer and catalytic activity. Oxygen vacancy concentration (δ) defines the density of active sites for polysulfide adsorption and redox mediation; higher δ (e.g., CeO_2_−_X_) promotes catalytic conversion but may reduce structural stability. Lewis acidity (pKa of [REE(H_2_O)_n_]^3+^) directly correlates with polysulfide binding strength; higher acidity (smaller pKa) leads to stronger adsorption, but excessive acidity causes passivation.

**FIGURE 14:**
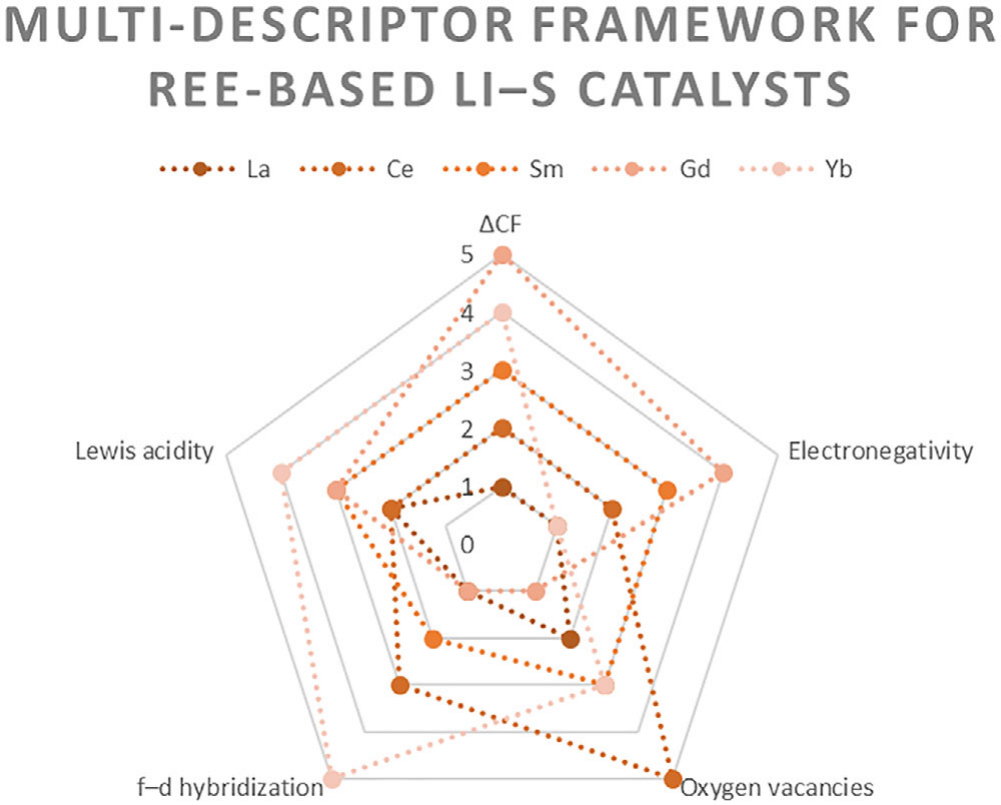
Shows a radar chart comparing five representative rare‐earth elements (La, Ce, Sm, Gd, Yb) across these five catalytic descriptors.

To visualize how these five descriptors vary across the lanthanide series, Figure 14 presents a radar chart comparing five representative rare‐earth elements (La, Ce, Sm, Gd, Yb). Scores (1–5) are normalized based on standard periodic trends and consensus from the inorganic chemistry literature. The chart reveals clear trade‐offs: Ce excels in oxygen vacancies but has moderate Lewis acidity; Gd exhibits strong crystal‐field splitting but very weak f–d hybridization; Yb combines strong f–d hybridization with high Lewis acidity but low electronegativity. This visualization supports the argument that no single REE optimizes all descriptors, and rational catalyst design requires multi‐parameter optimization.

### 7.2 Machine‐Learning‐Accelerated Discovery

The multi‐descriptor framework proposed above naturally lends itself to data‐driven and machine‐learning‐assisted catalyst design. Building on recent advances, we propose a three‐stage workflow for REE‐based Li–S catalysts. In Stage 1, high‐throughput DFT calculations compute adsorption energies (Li_2_S_4_, Li_2_S_2_, Li_2_S) and Li_2_S nucleation barriers for all 15 lanthanides on a standardized surface (e.g., N‐doped graphene or CeO_2_(111)) using a consistent computational protocol to ensure comparability. In Stage 2, the five descriptors (ΔCF, χ, Hfd, δ, pKa) are used as input features to train interpretable machine learning models (e.g., random forest or graph neural networks) that predict catalytic activity; the most influential descriptors are identified via SHAP analysis.

This workflow aligns with recent breakthroughs in the field. For instance, a study by researchers from Tsinghua University and Northwestern Polytechnical University developed a data‐driven framework that extracted universal structure–property relationships (UQSPRs) from a large‐scale, heterogeneous dataset spanning 20 years (2004–2024) and over 2900 peer‐reviewed studies. Their dataset contained 481 data points covering diverse transition metal compounds and five representative polysulfide species, enabling robust and generalizable model learning. They proposed a geometric descriptor (dispersion factor) that predicts catalytic activity, trained a collaborative model using random forests and feature screening, and screened 374 833 materials, experimentally validating CrB_2_ as a high‐performance catalyst [161].

**FIGURE 15:**
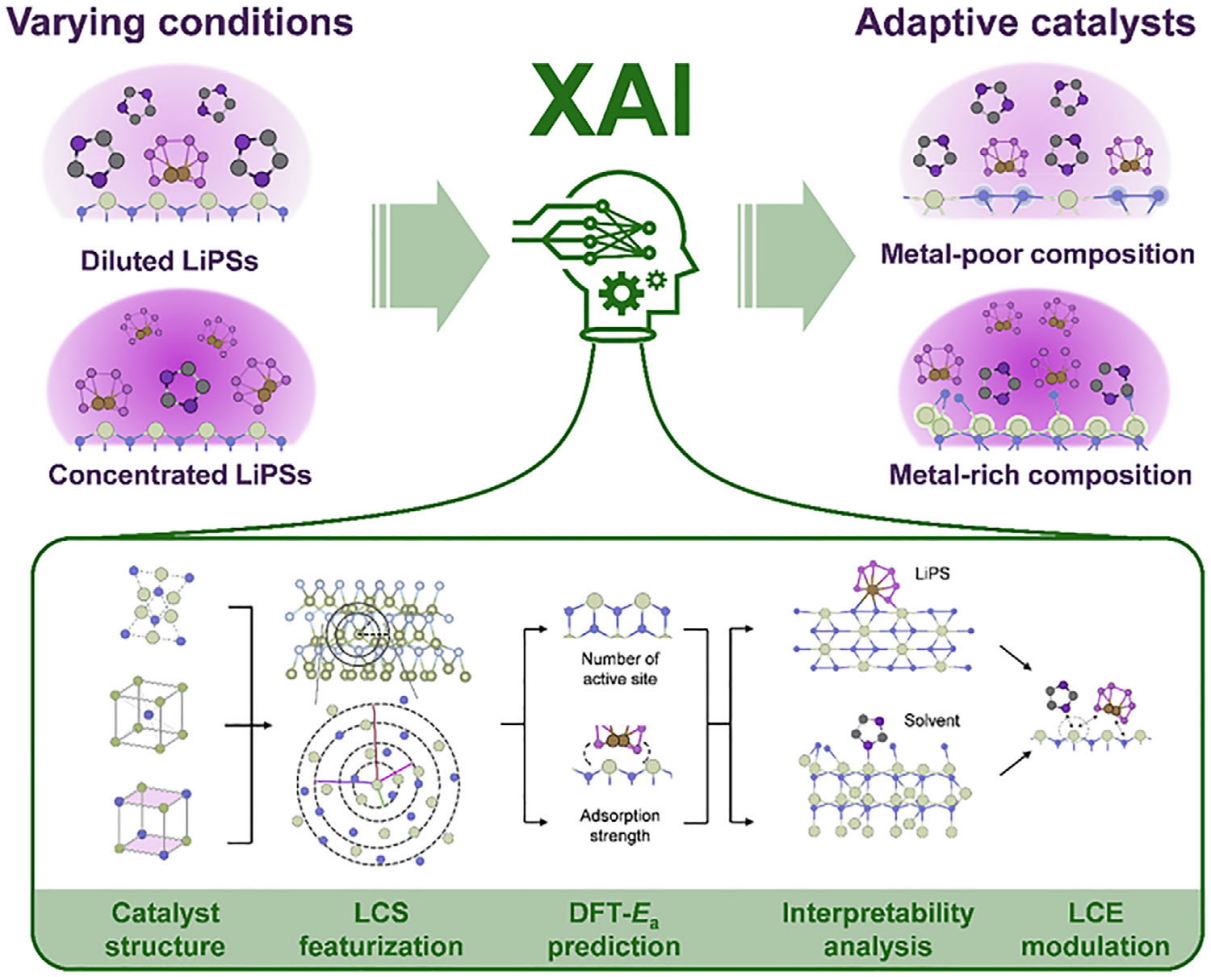
Schematic of XAI‐driven adaptive catalyst design for high‐performance Li‐S batteries under varying conditions [166]. Adapted with permission from Ref. [166]. Copyright 2025 Elsevier.

Similarly, an explainable‐AI‐based approach reported in a 2025 Patterns commentary intelligently designs catalysts adaptive to diverse local chemical environments in batteries, achieving exceptional catalytic and battery performance by concurrently considering intrinsic catalyst properties and extrinsic electrolyte environments [166]. As illustrated in Figure 15, the workflow begins by featurizing the local chemical environment (LCE), including variables such as LiPS concentration (diluted vs. concentrated), metal composition (metal‐poor vs. metal‐rich), solvent type, and catalyst structure.

These features are then processed through an explainable AI (XAI) model that performs DFT‐based activation energy prediction and interpretability analysis. The XAI model identifies key factors governing catalytic activity, such as adsorption strength and number of active sites, and uncovers mechanistic links between catalyst properties and electrolyte components. The final output is an adaptive catalyst tailored to the specific local chemical environment, enabling optimized performance under varying operating conditions (e.g., lean electrolyte, high sulfur loading).

This approach digitizes local chemical environment structures and properties, training XAI‐based models to uncover mechanistic links between catalysts and electrolyte components such as solvents and LiPSs. Additionally, another study used machine learning to predict the electrochemical performance of biomass‐derived carbon hosts in Li–S batteries, employing SHAP analysis to identify key factors such as biomass composition, carbon synthesis conditions, and current density, achieving high‐precision predictions with errors as low as 9.67% [169]. This workflow directly follows successful examples in the literature, such as the ML workflow for Li–S catalysts reported by Han et al. [163] and the descriptor‐importance ranking shown in their subsequent work [161].

### 7.3 High‐Entropy and Heterostructure Engineering

High‐entropy REE oxides offer a powerful platform to fine‐tune the multi‐descriptor landscape. By mixing multiple lanthanides at the A‐site, crystal‐field splitting and electronegativity can be continuously varied, achieving “optimal” polysulfide adsorption without extreme binding. The recent work by Zhou et al. on (La,Nd,Sm,Eu,Gd)_2_Zr_2_O_7_ pyrochlore exemplifies this approach: the authors showed that increasing the number of REE components systematically reduces crystal‐field splitting, weakens Zr–O covalency, and lowers Li_2_S_4_ binding to an ideal intermediate range [27]. A study by Liu et al. [40] on vacancy‐engineered ceria (Ov‐CeO_2_/CNT) demonstrates that oxygen vacancies dynamically modulate 4f‐orbital‐driven redox catalysis, enabling bidirectional sulfur conversion in Li–S batteries, which highlights that redox‐flexible rare‐earth catalysts featuring partially filled 4f orbitals enable orbital‐level modulation of sulfur electrochemistry. Future work should systematically map the composition descriptor performance relationship in these multi‐component systems. Heterostructures combining REE oxides with transition metals (e.g., Cu–CeO_2_−_x_@N/C) leverage interfacial f–d hybridization to enhance charge transfer and catalytic stability under lean electrolyte conditions. A study by Wang et al. [45] designed a TMs‐REOs heterojunction catalyst consisting of an N‐doped carbon shell containing embedded ultrafine Gd_2_O_3_ and Co nanocrystals (Gd_2_O_3_/Co@NC). Experimental and theoretical results revealed that the strong coupling between Co and Gd_2_O_3_ in a large number of heterojunctions endows the catalyst with moderate adsorption and satisfactory durability.

The cells assembled with a Gd_2_O_3_/Co@NC modified separator exhibited high rate capacity (628.0 mAh g^−1^ at 4C), cycling stability (504.2 mAh g^−1^ after 500 cycles at 2C), and sulfur utilization (4.8 mAh cm^−2^ under sulfur loading of 5.1 mg cm^−2^). This study highlights the invalidation mechanism of TMs in Li–S batteries and will inspire the design of advanced heterojunction catalysts through the coupling of TMs and REOs.

Similarly, Lai et al. [85] constructed a multicomponent La@Sn_3_O_4_/CeO_2_ heterostructure modified by rare‐earth elements. The unique 4f5d electronic orbitals and variable valence states of the rare‐earth elements effectively enhance the catalytic efficiency for polysulfides, exhibiting efficient sulfur utilization as well as good reaction kinetic rates. The La@Sn_3_O_4_/CeO_2_/S heterostructure composite cathode exhibited excellent initial discharge capacity (up to 1560 mAh g^−1^ at 0.1 C) and good cycling stability (0.09% decay rate over 500 cycles at 1 C). A study introduced a strategically engineered electrocatalyst, Cu‐CeO_2_−_x_@N/C, to enhance lean‐electrolyte lithium–sulfur battery performance. This catalyst, featuring in situ synthesized Cu clusters, regulates oxygen vacancies in CeO_2_ and forms Cu‐CeO_2_−_x_ heterojunctions, thereby diminishing sulfur conversion barriers and hastening reaction kinetics through the generation of S_3_^2^^−^/S_3_^−^ intermediates. The 1% Cu‐CeO_2_−x@N/C cell demonstrated robust performance, achieving an initial discharge capacity of 793.2 mAh/g at 5 C over 500 cycles and maintaining a capacity of 719.9 mAh/g at 0.3 C with an electrolyte‐to‐sulfur ratio of 5 µL mg^−1^ and a high sulfur loading of 5.4 mg cm^−2^ after 60 cycles [170].

High‐entropy oxides (HEOs) have also emerged as a powerful platform. A study reported a flower‐like HEO electrocatalyst composed of Bi, Sb, W, V, and Mo cations. The high‐density grain boundaries alongside the crystalline–amorphous heterophase structure provide abundant catalytically active centers, while strong d–p orbital coupling between 3d/4d/5d metals and p‐block metals strengthens chemical absorption and enhances catalytic activity. The HEO BiSbWVMoO‐modified separators achieved a high discharge capacity of 1440.4 mAh g^−1^ at 0.1C, a remarkable rate capability of 607.9 mAh g^−1^ at 5C, and a prolonged lifespan of over 1000 cycles at 1C with a low capacity decay of 0.053% per cycle [171].

### 7.4 Sustainability and Circular Economy

The long‐term viability of REE‐based Li–S batteries depend on minimizing material usage and enabling closed‐loop recycling. Light REEs (La, Ce, Nd) are relatively abundant ($2–5 kg^−1^ for La_2_O_3_) and can be deployed at scale. Heavy REEs (Gd, Yb, Lu) are scarce ($30–50 kg^−1^) and should be used only in atomically dispersed form (<0.1 wt.%). Recent advances in REE recovery include graphene oxide aerogels achieving >95% adsorption efficiency over multiple cycles [157], pseudocapacitive electrosorption with high selectivity for Nd^3+^ over Li^+^ and Mg^2+^ [158], and Co–FeS_2_@C composites for Yb^3+^ recovery with a capacity of 129.2 mg g^−1^ and excellent cycling stability [159]. A critical future direction is the integration of recovery functionality directly into battery design for example, using magnetic REE oxides or water‐soluble REE salts that can be easily leached at the end‐of‐life.

## Funding

This work was supported by the UK‐Engineering Physics and Science Research Council (Grant No. EP/S032886/1), the Scientific Research Project of Anhui Higher Education Institution (2024AH051843, 2024AH051839), and the Electrochemical Energy Storage and Catalysis Innovation Team.

## Conflicts of Interest

The authors declare no conflicts of interest.

## Data Availability

The data that support the findings of this study are available from the corresponding author upon reasonable request.
